# Comprehensive strategy improves the genetic diagnosis of different polycystic kidney diseases

**DOI:** 10.1111/jcmm.16608

**Published:** 2021-05-25

**Authors:** Hua‐Ying Hu, Jing Zhang, Wei Qiu, Chao Liang, Cun‐Xi Li, Tian‐Ying Wei, Zhan‐Ke Feng, Qing Guo, Kai Yang, Zu‐Guo Liu

**Affiliations:** ^1^ Department of Ophthalmology Xiang'an Hospital of Xiamen University Fujian Provincial Key Laboratory of Ophthalmology and Visual Science School of Medicine, Xiamen University Fujian Engineering and Research Center of Eye Regenerative Medicine Eye Institute of Xiamen University Xiamen China; ^2^ Jiaen Genetics Laboratory Beijing Jiaen Hospital Beijing China; ^3^ Prenatal Diagnosis Center Shijiazhuang Obstetrics and Gynecology Hospital Hebei China; ^4^ Department of Urology Beijing Friendship Hospital Capital Medical University Beijing China; ^5^ Department of Pediatric Orthopedics Shijiazhuang Obstetrics and Gynecology Hospital Hebei China; ^6^ Prenatal Diagnosis Center Beijing Obstetrics and Gynecology Hospital, Capital Medical University Beijing China

**Keywords:** long‐range PCR, *PKD1*, *PKHD1*, polycystic kidney disease, *TMEM107*, *TMEM67*, whole‐exome sequencing

## Abstract

Polycystic kidney disease (PKD) is known to occur in three main forms, namely autosomal dominant PKD (ADPKD), autosomal recessive PKD (ARPKD) and syndromic PKD (SPKD), based on the clinical manifestations and genetic causes, which are diagnosable from the embryo stage to the later stages of life. Selection of the genetic test for the individuals with diagnostic imaging reports of cystic kidneys without a family history of the disease continues to be a challenge in clinical practice. With the objective of maintaining a limit on the time and medical cost of the procedure, a practical strategy for genotyping and targeted validation to resolve cystogene variations was developed in our clinical laboratory, which combined the techniques of whole‐exome sequencing (WES), Long‐range PCR (LR‐PCR), Sanger sequencing and multiplex ligation–dependent probe amplification (MLPA) to work in a stepwise approach. In this context, twenty‐six families with renal polycystic disorders were enrolled in the present study. Thirty‐two variants involving four ciliary genes (*PKD1*, *PKHD1*, *TMEM67* and *TMEM107*) were identified and verified in 23 families (88.5%, 23/26), which expanded the variant spectrum by 16 novel variants. Pathogenic variations in five foetuses of six families diagnosed with PKD were identified using prenatal ultrasound imaging. Constitutional biallelic and digenic variations constituted the pathogenic patterns in these foetuses. The preliminary clinical data highlighted that the WES + LR PCR‐based workflow followed in the present study is efficient in detecting divergent variations in PKD. The biallelic and digenic mutations were revealed as the main pathogenic patterns in the foetuses with PKD.

## 1 INTRODUCTION

Hereditary polycystic kidney diseases (PKDs) exhibit clinical similarity and genetic diversity^1^ and may affect organ development and growth in patients ranging from embryos to adults.^2^ Polycystic kidney diseases originate from the cellular dysfunctions of chemo‐ and mechano‐sensations and fluid transport in renal tubules,^3^ which correlate with numerous molecules. Recent studies have delineated several genes associated with multiple signalling pathways to be involved in renal cystic disorders.^4^ The pathogenic variants in these genes could cause autosomal dominant/recessive polycystic kidney disease (ADPKD/ARPKD)^5^ and rare syndromes manifesting as renal polycysts.

Autosomal dominant polycystic kidney disease (ADPKD), which affects ~1 person among 1000 individuals, is the most common form of hereditary PKD and the underlying cause of end‐stage renal diseases (ESRD).^6^ The pathogenic variants in the *PKD1* and *PKD2* genes contribute to over 99% of all the ADPKD cases, half of which gradually progress to ESRD.^7^ The beginning time and the phenotypic severity of ADPKD depend on the nature of specific variants.^8^ The onset of ADPKD occurs in adulthood in the majority of the cases, while 2%‐5% of the cases exhibit early‐onset, even foetal‐onset, and the mechanism underlying early‐onset PKD has attracted great attention.^3^, ^9^ Recent in vivo and in vitro studies on biallelic and digenic mutations have proposed a ‘two‐hit’ model for cystogenesis in PKD, which involves the inactivation of both copies of a polycystic kidney disease gene by germline and somatic mutations, thereby leading to cyst formation.^10^

The *PKD1* gene comprises 46 exons within a 52‐kb locus. There are six highly homologous pseudogenes matching with the exons 1‐33,^11^ in addition to the high guanine‐cytosine (GC) content and simple repeats,^12^ which have rendered it challenging to analyse the genetic variations in the *PKD1* gene.

Autosomal recessive polycystic kidney disease (ARPKD) is mainly a result of the biallelic pathogenic variants in the *PKHD1* (PKD type 4) and *DZIP1L* (PKD type 5) genes, besides numerous syndromic PKD genes.^13^, ^14^ These syndromic disorders often exhibit severe early‐onset and multi‐systemic manifestations.^15^, ^16^

Advances in the next‐generation sequencing (NGS) technology have provided an opportunity to optimize the genetic diagnosis of PKDs for establishing their genotype‐phenotype correlations.^8^ Availability of adequate genetic tests would provide the patients with the benefit of precise counselling and management.^17^ In terms of feasibility, the gene panel test promises better sequencing depth,^18^ while the whole‐exome sequencing (WES) and whole‐genome sequencing (WGS) cover the full genome and allow the clinicians to identify novel pathogenic variants.^8^

In the present study, six cases of foetal‐onset PKD diagnosed using ultrasonography and 20 cases of adult‐onset PKD were recruited and registered for comprehensive genetic analyses using our new workflow. The overall detection rate observed reached 88.5%. The preliminary data showed that the WES + Targeted PKD1‐seq‐based workflow used in the present study was efficient in detecting the divergent mutations in different forms of PKD, particularly for the foetuses with polycysts.

## 2 MATERIALS AND METHODS

### 2.1 Participants and clinical analyses

The present study was approved by the Ethics Committee of Shijiazhuang Obstetrics and Gynecology Hospital (approval no.: 20200042). All participants were Chinese and were recruited from four medical centres (affiliate 2‐5) in the northern region of China. Informed consent for participation in the study was obtained from all recruits. A total of 46 individuals from 26 unrelated families with no consanguineous relationship were enrolled, among which 21 families were associated with adult‐onset PKDs, six families were associated with early‐onset PKDs, and one family was associated with both. The diagnostic criteria for adult‐onset PKD were according to the KDIGO guideline.^19^

### 2.2 Workflow set‐up for genetic analyses

**FIGURE 1:**
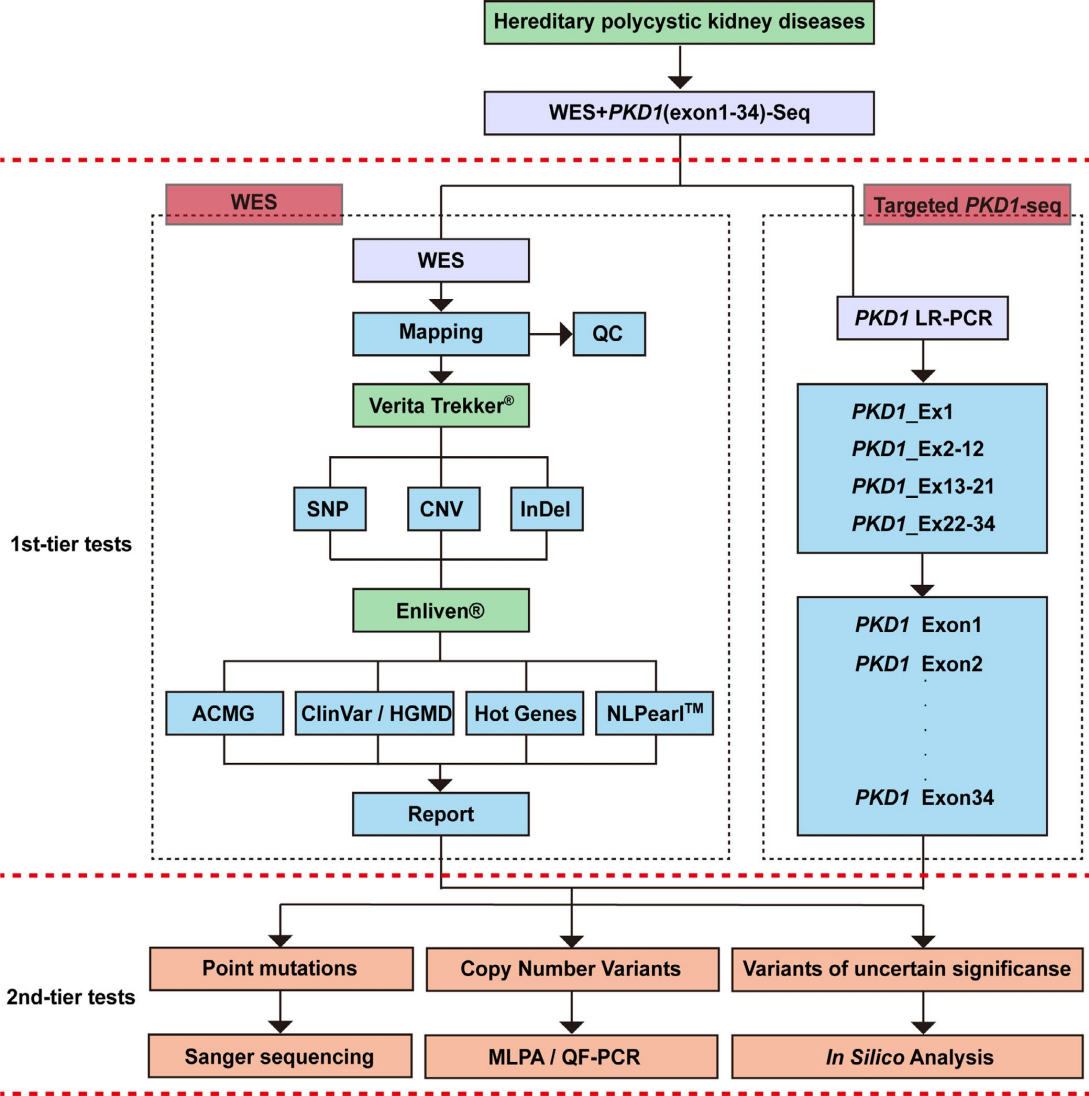
The diagnostic workflow for mutation analysis in PKD patients. The proposed complete strategy comprised three stepwise analyses. The first tier of tests were administrated simultaneously, which included whole‐exome sequencing (WES) and targeted PKD1 (exon 1‐34) sequencing. The targeted Sanger sequencing procedure was performed subsequent to long‐range PCR (LR‐PCR) that was performed to amplify the repetitive sequences in the four fragments spanning 1‐34 exons and introns as indicated in the right dashed box. WES was applied to screen single nucleotide polymorphisms (SNPs), insertions/deletions (InDels) and copy number variations (CNVs). When the first tier of tests identified a variant of unknown significance (VUS), in silico analysis was conducted to prognosticate its functional influence. When WES detected a microdeletion/microduplication mutation or a no point mutation, QF‐PCR and MLPA were performed, respectively. The ‘pathogenic’ and ‘likely pathogenic’ point mutations detected using WES were validated using Sanger sequencing as the second‐tier test. ACMG, American College of Medical Genetics and Genomics; HGMD, Human Gene Mutation Database; NL pearl™, Natural Language Pearl processing and analysis system by Berry Genomics, Inc

In view of the clinical and genetic heterogeneities of PKDs, the experiment cost and the analytical complexity of the *PKD1* gene, a comprehensive workflow combining the techniques of WES, targeted LR‐PCR plus sequencing, MLPA (multiplex ligation‐dependent probe amplification), QF‐PCR (quantitative fluorescence PCR) and in silico analysis was established, as illustrated in Figure 1. WES and targeted *PKD1*‐seq were applied simultaneously as the first tier of tests. The targeted sequencing was aimed to detect variants in the *PKD1* exons 1‐34, while WES aimed to detect variants in the genome‐wide coding region.

Sanger sequencing, MLPA, QF‐PCR and in silico analysis formed the second tier of tests aiming for mutation validation and functional prediction. All WES‐detected variants suspected with pathogenicity were verified using Sanger sequencing. The patients with no sequence variant were screened for possible rearrangements and/or CNVs in *PKD1* using MLPA and QF‐PCR. In this manner, the maximum turnaround time could be limited to just 2 weeks.

### 2.3 DNA extraction

Genomic DNA was extracted from the peripheral blood (for adults) and cord blood (for foetuses) samples (200 µL each) by using the DNA Blood Midi/Minikit (QIAGEN, Hilden, Germany) in accordance with the manufacturer's protocol.

### 2.4 Whole‐exome sequencing (WES)

Whole‐exome sequencing was performed on the probands in each family as described in a previous study.^20^ The details of the analysis procedures are provided in Figure 1 (left dashed block). The obtained DNA samples (1 µg/each) were subjected to quality evaluation using agarose gel electrophoresis and UV spectrophotometry, after, which the DNA fragments were hybridized and captured using the IDT’s xGen Exome Research Panel 2.0 (Integrated DNA Technologies, San Diego) according to the manufacturer's protocol. The libraries were screened for enrichment using qPCR, and the size distribution and concentration were determined using Agilent Bioanalyzer 2100 (Agilent Technologies, Santa Clara, CA). The Novaseq6000 platform (Illumina, San Diego) was employed for the genomic sequencing of ~300 pmol·L^−1^ of DNA per sample using Novaseq Reagent Kit.^21^ The raw reads (quality level Q30 > 89%) were aligned to the human reference genome (hg19/GRCh37) using the Burrows‐Wheeler Aligner tool, and the PCR duplicates were removed using Picard v1.57.^22^ Variant calling was performed using the Verita Trekker^®^ Variants Detection system v 2.0 (Berry Genomics, Inc, Beijing) and the Genome Analysis Toolkit.^23^ The SNPs and InDels with a frequency of >0.01 in 1000 genomes, ExAC and gnomAD_exomes were removed. The non‐synonymous variants were evaluated using Revel (an ensemble method for predicting the pathogenicity of missense variants using the following tools individually: MutPred, FATHMM, VEST, PolyPhen, SIFT, PROVEAN, MutationAssessor, MutationTaster, LRT, GERP, SiPhy, phyloP and phastCons).^24^ The variants were then annotated and interpreted using ANNOVAR (v 2.0) and Enliven^®^ Variants Annotation Interpretation systems (Berry Genomics, Inc, Beijing)^25^ according to the guidelines provided by the American College of Medical Genetics and Genomics (ACMG).^26^ A total of 313 genes associated with different polycystic kidney diseases were focused on and analysed (Table S1).

### 2.5 LR‐PCR for targeted *PKD1*‐seq

Long‐range PCR was performed to isolate the sequence of the *PKD1* exons 1‐34 from the other six pseudogenes (Figure 1, right dashed block). The primer information details are summarized in Table S2, and the PCR reaction conditions are provided in Table S3.

### 2.6 Sanger sequencing

Sanger sequencing was performed for the sequence analysis of exons 1‐34 of the *PKD1* gene (Figure 1, right dashed block; amplification products of LR‐PCR in Section 2.5) as well as for the validation of suspected variants in any of the genes detected using WES (Section 2.4).

### 2.7 Multiplex ligation–dependent probe amplification (MLPA)

In order to directly identify the CNVs and validate the findings of WES suggesting an exonic CNV of *PKD1,* MLPA was performed using the SALSA kit P351 (MRC‐Holland, Inc, Amsterdam) according to the manufacturer's protocol.

### 2.8 In silico analysis of conservatism and molecular modelling

All the missense variants identified in the present study were analysed for evolutionary conservatism of the affected amino acid residues using MEGA7 with default parameters.^27^

In the case of missense variants located in the well‐defined peptide structure models available in the PDB database,^28^ the Modeller 9V17 software was employed to predict the functional influence of the structural anomaly.^29^ In the case of missense variants not matching with any well‐defined peptide structure models available in the PDB database, the UniProt database^30^ was searched for a functional prediction using the Rosetta CM program as described in a previous study.^31^ The confidence threshold was set at ≥0.6.

In the molecular dynamics (MD) analysis, CHARMM22 was employed to add hydrogen atoms and N‐ and C‐terminal patches to the models.^32^ The generated models were solvated and neutralized with TIP3P water within a box at a minimum distance of 13 Å between the model and the wall of the box. All simulations were run using NAMD 2.9 and by applying periodic boundary conditions (PBC). The temperature was maintained at 300 K, and the pressure was maintained at 1 atm. The time step was set to 2 fs, the particle‐mesh Ewald method was applied to model the electrostatics, and the van der Waals interactions threshold was set at 12 Å. Both models included a three‐step pre‐equilibration totalling 600 ps, the last snapshots of which were selected as the beginning structures for 20‐ns productive simulations without constraints.

## 3 RESULTS

### 3.1 Clinical findings

Twenty‐six families were recruited in the present study based on a definitive polycystic imaging diagnosis. There were 46 participants from 16 pedigrees with a clear ADPKD family history and 10 families with PKD proband only. Hepatic cysts were detected in 19 (73.08%, 19/26) families. Twenty‐seven individuals from 22 adult‐onset PKD families had manifested symptoms and were diagnosed at the mean age of 30 years (range 20‐51). Twelve patients with ESRD were operated on for nephrectomy and pathological biopsy.

Six cases of in utero‐onset PKD were diagnosed using ultrasonography, one of which had a known family history of the disease (Family 22). The average gestational age at diagnosis was 22 weeks. Details of the clinical information of all the recruited patients are summarized in Table 1. In order to better illustrate the data from the PKD families, pedigree diagrams, imaging graphs, genetic variants, and pathological results were constructed into composite figures (Figures S1 and S2; 3).

**TABLE 1:** Clinical data from the probands and key family members of all families

Family No.	ID	Age (y)	Gender	Diagnosed age/Initial indication	Family history of PKD	Renal cysts	Hepatic cysts	Hypertension (Age)	Renal osteopathy	ESRD (Age)	Other symptoms	Operations
1	1.1	55	M	40/haematuria	(+)	(+)	(+)	(+)	(+)	(+) (47)	Chronic cardiac insufficiency, coronary arteriosclerosis, cirrhosis	Left nephrectomy (2004); KT (2017)
2	2.1	49	F	25/imaging diagnosis	(−)	(+)	(−)	(+)	(+)	(+) (41)	/	Left nephrectomy (2012)
3	3.1	55	M	23/imaging diagnosis	(−)	(+)	(+)	(+)	(+)	(+) (51)	Coronary heart disease, hyperkalemia	Right nephrectomy and KT (2017)
4	4.1	55	M	24/haematuria	(+)	(+)	(+)	(+)	(+)	(+) (52)	/	Right nephrectomy (2018); KT (2019)
	4.2	67^a^	F	32/haematuria	(+)	(+)	(+)	(+)	(+)	(+) (55)	/	Died at 67 y old
5	5.1	40	M	38/pruritus, oliguria	(−)	(+)	(+)	(+)	(+)	(+) (39)	Hypertensive cardiopathy, SHPT	Left nephrectomy and KT (2018)
6	6.1	55	F	30/imaging diagnosis	(+)	(+)	(+)	(+)	(+)	(+) (52)	Right breast cancer (radical mastectomy)	Right nephrectomy and KT (2018)
7	7.1	53	F	51/pruritus, osphyalgia	(+)	(+)	(+)	(+)	(+)	(+) (52)	Pituitary adenoma (postoperation)	Left nephrectomy and KT (2018)
8	8.1	48	F	20/imaging diagnosis	(+)	(+)	(+)	(+)	(+)	(+) (44)	Left clavicle fracture (postoperation)	Right nephrectomy (2017); KT (2018)
9	9.1	36	M	25/imaging diagnosis	(+)	(+)	(+)	(+)	(−)	(−)	/	/
10	10.1	40	M	30/imaging diagnosis	(+)	(+)	(+)	(+)	(−)	(−)	SHPT	/
	10.2	10	F	/		(−)	(−)	(−)	(−)	(−)	/	
11	11.1	30	M	27/imaging diagnosis	(+)	(+)	(+)	(+)	(−)	(−)	/	/
12	12.1	35	M	25/haematuria	(−)	(+)	(+)	(+)	(−)	(−)	/	/
13	13.1	30	M	30/imaging diagnosis	(+)	(+)	(−)	(+)	(−)	(−)	/	/
14	14.1	30	M	27/haematuria	(+)	(+)	(+)	(+)	(−)	(−)	/	/
15	15.1	31	M	28/imaging diagnosis	(+)	(+)	(+)	(+)	(−)	(−)	/	/
	15.2	59	M	35/haematuria		(+)	(+)	(+)	(+)	(+) (44)	Hypertensive cardiopathy	Left nephrectomy (2018); KT (2019)
16	16.1	26	M	24/haematuria	(+)	(+)	(+)	(+)	(−)	(−)	/	/
	16.2	52	M	30/haematuria	(+)	(+)	(+)	(+)	(+)	(−)	/	/
17	17.1	28	M	26/haematuria	(+)	(+)	(+)	(+)	(−)	(−)	Pancreatic cysts	/
18	18.1	42	M	37/imaging diagnosis	(−)	(+)	(−)	(+)	(−)	(−)	/	/
19	19.1	61	M	50/haematuria	(+)	(+)	(+)	(+)	(+)	(+) (55)	SHPT; myocardial infarction	Left nephrectomy (2017)
20	20.1	50	F	30/imaging diagnosis	(−)	(+)	(+)	(+)	(+)	(+) (47)	Cholecystotomy; Appendectomy	Left nephrectomy and KT (2018)
21	21.1	27	F	25/imaging diagnosis	(+)	(+)	(+)	(+)	(−)	(−)	/	/
	21.2	54	F	30/imaging diagnosis		(+)	(+)	(+)	(+)	(+) (49)	Hypertensive cardiopathy, SHPT	Left nephrectomy (2019)
	21.3	28	M	/		(−)	(−)	(−)	(−)	(−)	/	/
	21.4	/	/	In utero/imaging diagnosis		(+)	(−)	/	/	/	/	Selective abortion (22nd GW)
	21.5	/	/	/		(−)	(−)	/	/	/	/	/
22	22.1	0	/	In utero/imaging diagnosis	(+)	(+)	(+)	/	/	/	/	Selective abortion (24th GW)
	22.2	33	F	/		(−)	(−)	(−)	(−)	(−)	/	/
	22.3	34	M	34/imaging diagnosis		(+)	(−)	(−)	(−)	(−)	/	/
	22.4	60	M	39/haematuria		(+)	(+)	(+)	(−)	(−)	/	/
	22.5	61	F	/		(−)	(−)	(−)	(−)	(−)	/	/
23	23.1	0	/	In utero/imaging diagnosis	(−)	(+)	(−)	/	/	/	/	Selective abortion (24th GW)
	23.2	30	F	/		(−)	(−)	(−)	(−)	(−)	/	/
	23.3	31	M	/		(−)	(−)	(−)	(−)	(−)	/	/
24	24.1	0	/	In utero/imaging diagnosis	(−)	(+)	(−)	/	/	/	Bladder dysplasia; oligohydramnios	Selective abortion (23rd GW)
	24.2	27	F	/		(−)	(−)	(−)	(−)	(−)	/	/
	24.3	26	M	/		(−)	(−)	(−)	(−)	(−)	/	/
25	25.1	0	/	In utero/imaging diagnosis	(−)	(+)	(−)	/	/	/	Polydactylism; occipital bulge; abnormal foot position; oligohydramnios	Selective abortion (24th GW)
	25.2	30	F	/		(−)	(−)	(−)	(−)	(−)	/	/
	25.3	32	M	/		(−)	(−)	(−)	(−)	(−)	/	/
26	26.1	0	/	In utero/imaging diagnosis	(−)	(+)	(−)	/	/	/	Cerebellar hypoplasia; bilateral choroid plexus cysts	Selective abortion (22nd GW)
	26.2	29	F	/		(−)	(−)	(−)	(−)	(−)	/	/
	26.3	30	M	/		(−)	(−)	(−)	(−)	(−)	/	/

Abbreviations: ESRD, end‐stage renal disease; F, female; GW, gestational week; KT, kidney transplant; M, male; SHPT, secondary hyperparathyroidism.

^a^ decease age.

### 3.2 Identification of gene variants in PKD families

**FIGURE 2:**
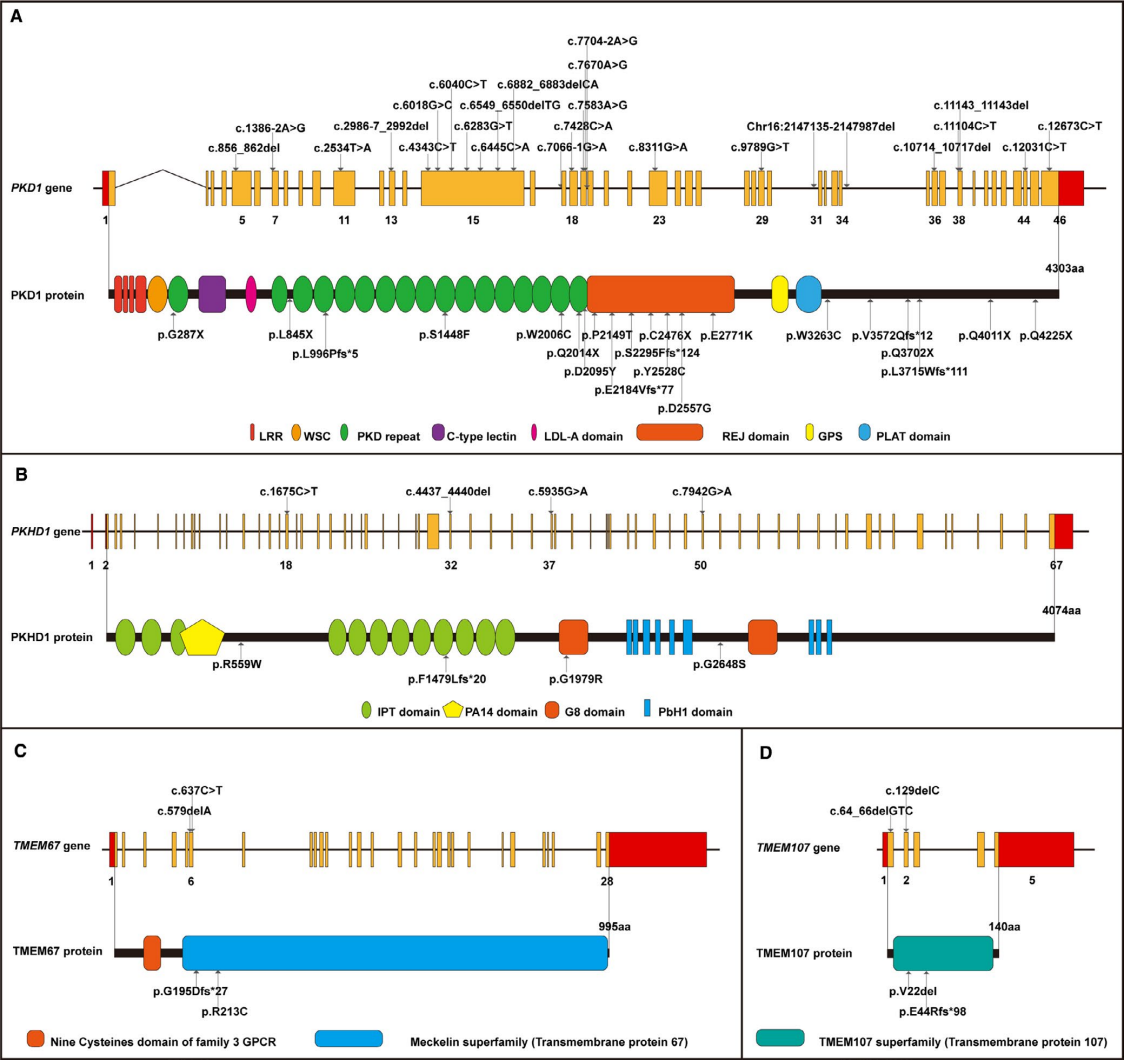
All diagnostic variants were illustrated at the gene exon and protein domain levels. The exon number with the mutation is labelled below the yellow bars. The protein domains are depicted in different colours. A, Graphic view of the 24 variants across the full length of the *PKD1* gene. B, Graphic view of the four variants across the *PKHD1* gene. C, Graphic view of the two variants in the *TMEM67* gene. D, Graphic view of the two novel variants in the *TMEM107* gene

A total of 32 variants were identified in 23 (88.5%, 23/26) families, which were associated with four cystogenes, namely *PKD1* (MIM *601313, 24 variants), *PKHD1* (MIM *606702, 4 variants), *TMEM67* (MIM *609884, 2 variants) and *TMEM107* (MIM *616183, 2 variants). Among the identified variants, 17 variants (53.12%) were novel variants and 15 variants (46.88%) were the previously described ones. According to the ACMG evidence, 6.25% of the variants were pathogenic (P), 59.38% of the variants were likely pathogenic (LP), and 34.37% of the variants were variants of uncertain significance (VUS). In resolved families, 75% of the variants were autosomal dominant (AD) and 25% were autosomal recessive (AR). All the variants detected in the present study are listed in Figure 2 and Table 2.

**TABLE 2:** All the diagnostic variants identified in the present study

F_No.	ID	Gene^①^	Exon/Intron	DNA variant	Protein variant	Variation frequencies in 3 databases^②^	NMD_predict	Revel_Score^③^	pLI^④^	Database^⑤^	PMID^⑥^	Level (Evidence)^⑦^
1	1.1	*PKD1*	Exon18	c.7428C > A	p.C2476X	0; 0; 0	YES, 0.58	–	0.999905332	Novel	–	LP (PVS1 + PM2)
2	2.1	*PKD1*	Exon36	c.10714_10717delGTCT	p.V3572Qfs^*^12	0; 0; 0	YES, 0.83	–	0.999905332	Novel	–	LP (PVS1 + PM2)
3	3.1	*PKD1*	Chr16:2147135‐2147987del									P
4	4.1	*PKD1*	Exon15	c.6445C > A	p.P2149T	0; 0; 0	NO	0.649	0.999905332	Novel	–	VUS (PM1 + PM2)
	4.1	*PKD1*	Exon38	c.11143_11143delC	p.L3715Wfs^*^111	0; 0; 0	YES, 0.89	–	0.999905332	HGMD/PKDB	–	LP (PVS1 + PM2)
5	5.1	*PKD1*	Exon23	c.8311G > A	p.E2771K	0; 0; 0	NO	0.521	0.999905332	HGMD/PKDB	11115377	VUS (PM1 + PM2)
6	6.1	*PKD1*	Intron16	c.7066‐1G > A		0; 0; 0	YES, 0.55	–	0.999905332	Novel	–	LP (PVS1 + PM2)
7	7.1	*PKD1*	Exon11	c.2534T > A	p.L845X	0; 0; 0	YES, 0.20	–	0.999905332	Novel	–	LP (PVS1 + PM2)
8	8.1	*PKD1*	Exon5	c.856_862delTCTGGCC	p.G287X	0; 0; 0	YES, 0.07	–	0.999905332	HGMD	21694639	LP (PVS1 + PM2)
9	9.1	*PKD1*	Exon13	c.2986‐7_2992del CCTGCAGCTGACGG	p.L996Pfs^*^5	0; 0; 0	YES, 0.23	–	0.999905332	Novel	–	LP (PVS1 + PM2)
10	10.1; 10.2	*PKD1*	Exon19	c.7670A > G	p.D2557G	0; 0.000092; 3.87241e‐05	NO	0.931	0.999905332	PKDB	–	VUS (PM1 + PM2 + PP3)
	10.1; 10.2	*PKD1*	Intron19	c.7704‐2A > G		0; 0; 0	YES, 0.60	NA	0.999905332	Novel	–	LP (PVS1 + PM2)
11	11.1	*PKD1*	Exon15	c.6549_6550delTG	p.E2184Vfs^*^77	0; 0; 0	YES, 0.53	NA	0.999905332	Novel	–	LP (PVS1 + PM2)
12	12.1	*PKD1*	Exon15	c.6040C > T	p.Q2014X	0; 0; 0	YES, 0.47	NA	0.999905332	HGMD/PKDB	12007219	LP (PVS1 + PM2)
13	13.1	*PKD1*	Exon15	c.6018G > C	p.W2006C	0; 0; 0	NO	0.943	0.999905332	HGMD/PKDB	24611717	LP (PM1 + PM2 + PS1 + PP3)
14	14.1	*PKD1*	Exon15	c.6882_6883delCA	p.S2295Ffs^*^124	0; 0; 0	YES, 0.56	–	0.999905332	Novel	–	LP (PVS1 + PM2)
15	15.1; 15.2	*PKD1*	Exon38	c.11104C > T	p.Q3702X	0; 0; 0	YES, 0.86	–	0.999905332	HGMD/PKDB	12220456	LP (PVS1 + PM2)
16	16.1; 16.2	*PKD1*	Exon44	c.12031C > T	p.Q4011X	0; 0; 0	YES, 0.93	–	0.999905332	HGMD/PKDB	9521593	P (PVS1 + PM2 + PP4)
	16.1; 16.2	*PKD1*	Exon15	c.6283G > T	p.D2095Y	0; 0; 0	NO	0.545	0.999905332	Novel	–	VUS (PM1 + PM2 + PP4)
17	17.1	*PKD1*	Exon46	c.12673C > T	p.Q4225X	0; 0; 0	NO, 0.98	–	0.999905332	HGMD	23300259	LP (PVS1 + PM2)
18	18.1	*PKD1*	Exon29	c.9789G > T	p.W3263C	0; 0; 0	NO	0.73	0.999905332	PKDB	–	VUS (PM2)
21	21.3; 21.4; 21.5	*PKD1*	Intron6	c.1386‐2A > G		0; 0; 0	YES, 0.11	–	0.999905332	Novel	–	LP (PVS1 + PM2)
	21.1; 21.2; 21.4	*PKD1*	Exon19	c.7583A > G	p.Y2528C	0; 0; 0	NO	0.898	0.999905332	Novel	–	VUS (PM1 + PM2 + PP3)
22	22.1; 22.3; 22.4	*PKD1*	Exon15	c.4343C > T	p.S1448F	0.000199681; 0.000109; 6.93368e‐05	NO	0.284	0.999905332	HGMD	27567292	VUS (PM1)
	22.1; 22.3; 22.5	*PKHD1*	Exon50	c.7942G > A	p.G2648S	0.00479233; 0.004197; 0.00564584	NO	0.357	2.89E‐23	HGMD	24162162	LB (with deep concern)
	22.1; 22.2	*PKHD1*	Exon18	c.1675C > T	p.R559W	0.00698882; 0.00135589; 0.00154668	NO	0.17	2.89E‐23	Novel	–	LB (with deep concern)
23	23.1; 23.2	*PKHD1*	Exon32	c.4437_4440delCATA	p.F1479Lfs^*^20	0; 0; 0	YES, 0.36	–	2.89E‐23	Novel	–	LP (PVS1 + PM2)
	23.1; 23.3	*PKHD1*	Exon37	c.5935G > A	p.G1979R	0; 0; 0	NO	0.636	2.89E‐23	HGMD	22882926	VUS (PM1 + PM2)
24	24.1; 24.3	*TMEM67*	Exon6	c.637C > T	p.R213C	0; 0; 0	NO	0.759	1.21E‐17	HGMD	17160906	VUS (PM2 + PM3 + PP3)
	24.1; 24.2	*TMEM67*	Exon6	c.579delA	p.G195Dfs^*^27	0; 0; 4.06204e‐06	NO	–	1.21E‐17	HGMD	17377820	LP (PVS1 + PM2)
25	25.1; 25.3	*TMEM107*	Exon2	c.129delC	p.E44Rfs^*^98	0; 0; 0	NO	–	–	Novel	–	LP (PVS1 + PM2)
	25.1; 25.2	*TMEM107*	Exon1	c.64_66delGTC	p.V22del	0; 0; 0	NO	–	–	Novel	–	LP (PM2 + PM3 + PM4)

Note

F_No.: family_number; M_No.: mutant_number; ①Transcript ID: *PKD1*: *Polycystic Kidney Disease 1* (NM_001009944.2); *PKHD1*: *Polycystic Kidney and Hepatic Disease 1* (NM_138694.3); *TMEM67*: *Transmembrane protein 67* (NM_153704.6); *TMEM107*: *Transmembrane protein 107* (NM_032354.3); ②1000 genomes (https://www.internationalgenome.org/); ExAC (http://exac.broadinstitute.org); gnomAD_exomes (http://gnomad.broadinstitute.org/); ③An ensemble method for predicting the pathogenicity of missense variants on the basis of individual tools: MutPred, FATHMM, VEST, PolyPhen, SIFT, PROVEAN, MutationAssessor, MutationTaster, LRT, GERP, SiPhy, phyloP, and phastCons (http://dx.doi.org/10.1016/j.ajhg.2016.08.016). ④pLI: https://gnomad.broadinstitute.org/ ⑤HGMD^®^: Human Gene Mutation Database (Professional Version 2019.4); PKDB: Polycystic Kidney Disease Database (https://pkdb.mayo.edu/) ⑥PMID: PubMed ID (https://pubmed.ncbi.nlm.nih.gov/) ⑦ACMG: The American College of Medical Genetics and Genomics; P: pathogenic; LP: likely pathogenic; VUS: variants of unknown significance; LB: likely benign.

The detection rate for the patients with phenotypically manifested adult‐onset PKDs was 90.9% (20/22). Twenty‐four different variants were detected in the *PKD1* gene, including 12 truncations, 3 splicing variants, 8 missense and 1 exon deletion, among which 13 variants were reported for the first time (Table 2). Double mutations in a single allele of the *PKD1* gene were detected in families 4, 10 and 16 (Figure [Link], [Link], [Link], [Link]).

**FIGURE 3:**
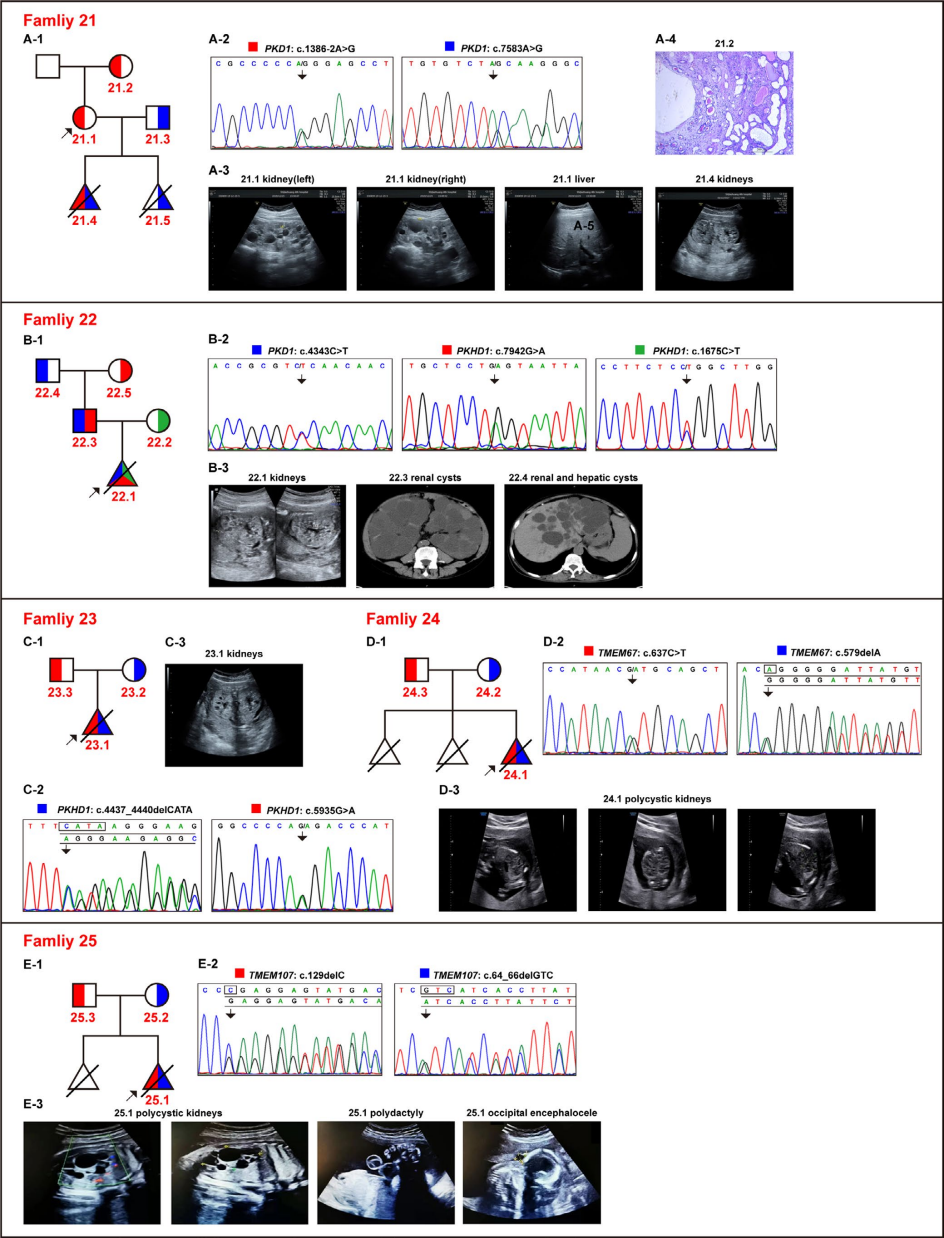
Pedigree diagrams, clinical findings and genetic variants for families 21‐25. (A‐1, B‐1, C‐1, D‐1, E‐1) The pedigree diagrams for families 21, 22, 23, 24 and 25, respectively. Traits carried by the parents are displayed in different colours. (A‐2, B‐2, C‐2, D‐2, E‐2) The detected variations in each family are depicted in sequence and peak patterns, either in forward or in reverse complementary sequence. Different coloured blocks of the variants in the pedigrees corresponded to the parents they were inherited from. (A‐3, B‐3, C‐3, D‐3, E‐3) The representative images of clinical ultrasonography or the computed tomography of X‐rays for the probands or the key patients in the respective families. (A‐4) The H&E staining micrograph demonstrated that polycystic tubule dilation was apparent in the kidney tissue of Patient 21.2

Specimens from 6 foetuses with PKD diagnosis were evaluated (Figure 3), which revealed 11 pathogenic variants, including 6 novel ones, in 5 foetuses (83.3%, 5/6). These 5 typical cases are described ahead in detail.

The couple from Family 21 had undergone abortion due to foetal PKD twice prior to the present pregnancy. Both the aborted foetuses carried the compound heterozygous pathogenic variants, namely *PKD1*: c.1386‐2A > G and *PKD1*: c.7583A > G (p.Y2528C) from each parent, and these variants were inferred to have contributed to the foetal PKD.^33^

Family 22 presented a complex case with three pathogenic alleles in the parents, among which the *PKD1*: c.4343C > T (p.S1448F) variant was a known pathogenic variant for adult‐onset manifestation, while the foetal PKD (22.1) was inferred to have been caused by the compound heterozygous variants *PKHD1*: c.1675C > T (p.R559W) and *PKHD1*: c.7942G > A (p.G2648S), which were inherited from the mother and the father, respectively (Figure 3).

Whole‐exome sequencing revealed that the foetal PKD proband in Family 23 had compound heterozygous variants in *PKHD1,* which was subsequently verified through Sanger sequencing. The mother and the father were asymptomatic carriers of *PKHD1*: c.4437_4440delCATA (p.F1479Lfs*20) and *PKHD1*: c.5935G > A (p.G1979R), respectively (Figure 3).

The foetuses in Family 24 and Family 25 appeared to have additional symptoms besides PKD. The couple from Family 24 had undergone abortion three times due to phenotypes similar to those in renal cystic disorders, bladder dysplasia and oligohydramnios. The sample of the third pregnancy was subjected to genetic testing using WES plus Sanger sequencing. The compound heterozygous variants of *TMEM67*: c.637C > T (p.R213C) and *TMEM67*: c.579delA (p.G195Dfs*27) were identified and likely agreed with the Meckel Gruber syndrome type 3 (MKS3, MIM #607316), which segregated from the asymptomatic parents (Figure 3). Another in utero*‐*onset proband in Family 25 manifested additional clinical appearances, including kidney cysts, polydactyly, occipital bulge, abnormal foot posture and oligohydramnios, as detected in ultrasonic imaging. The novel compound heterozygous variants *TMEM107*: c.64_66delGTC (p.V22del) and *TMEM107*: c.129delC (p.E44Rfs*98) were detected in *TMEM107* (causative for MKS type 13, MIM #617562) and were validated in time. These variants were inherited from asymptomatic parents (Figure 3).

### 3.3 Conservatism and molecular modelling analysis

In the present study, 12 missense variants were detected. It was demonstrated that all the affected amino acid residues were evolutionarily conserved across species (Figure S3).

**FIGURE 4:**
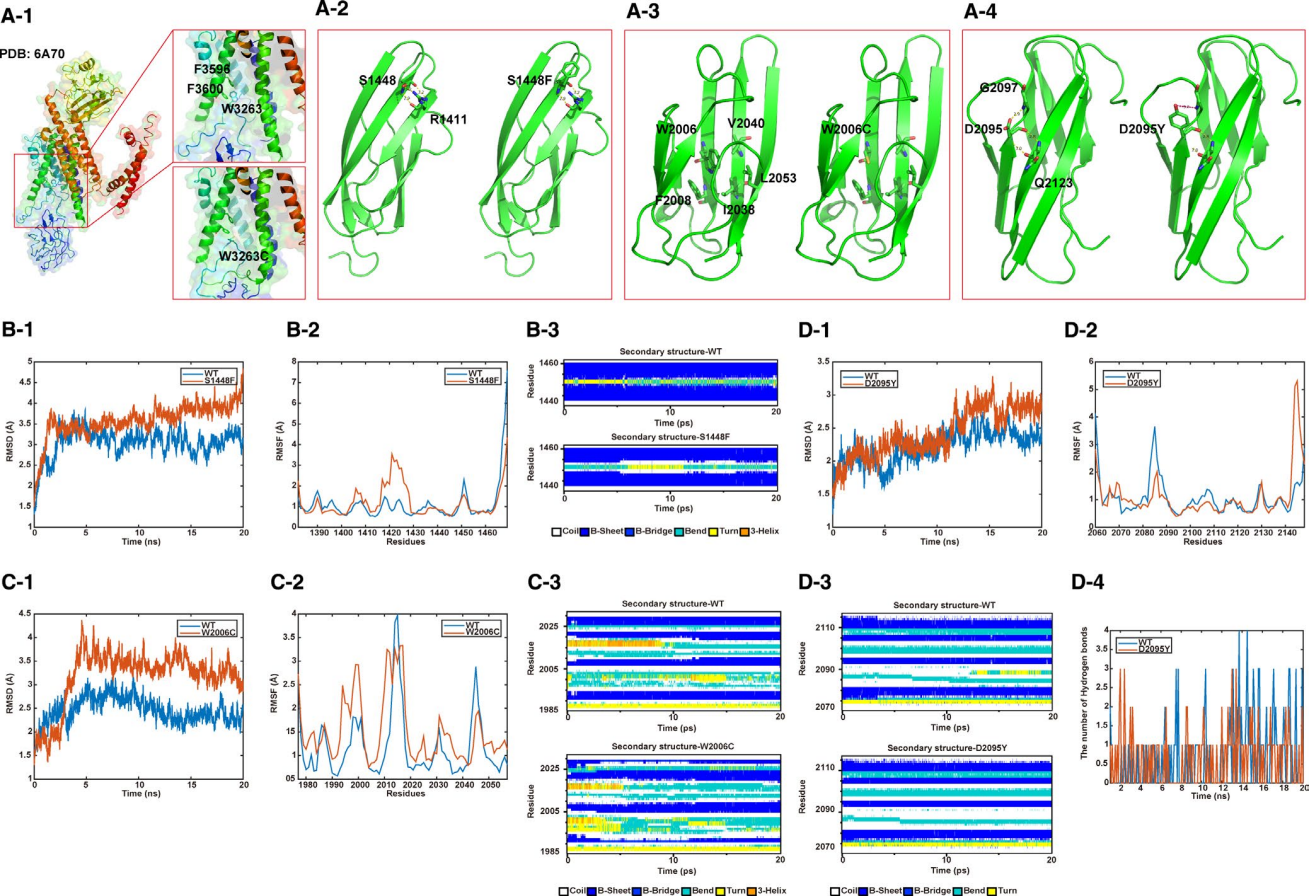
Molecular modelling analysis of 4 missense variants in the *PKD1* gene using the CHARMM22 program. (A‐1) The structural changes in PKD1: p.W3263C. The wild‐type structure is displayed in the upper box and the p.W3263C mutant structure is depicted in the lower box. (A‐2, A‐3, A‐4) The structural prediction of PKD1: p.S1448F, p.W2006C and p.D2095Y. Left side images illustrate the wild‐type structures, while their right side images illustrate the alternative interactions of the mutants. (B‐1, C‐1, D‐1) and (B‐2, C‐2, D‐2) RMSD and RMSF results for S1448F, W2006C and D2095Y contrasted with their wild types. (B‐3, C‐3, D‐3) Differences among the secondary structures of S1448F, W2006C and D2095Y contrasted with their wild types. (D‐4) Wild type D2095 and mutant D2095Y formed hydrogen bonds in different numbers

*PKD1*: c.9789G > T (p.W3263C) detected in Family 18 was the only variant that had a corresponding sequence structure indexed in the PDB database (no. 6A70).^34^ The molecular modelling results indicated that Trp^3263^ in the pre‐second transmembrane helix loop interacted with the Phe^3596^ and Phe^3600^ residues of the fifth transmembrane helix. This hydrophobic core appeared to stabilize the overall conformation of the pre‐second transmembrane helix loop, which may, in turn, stabilize the structure of the N‐terminal transmembrane domain. In addition, the p.W3263C replacing a large amino acid with benzene rings via a polar cysteine expectantly destroyed the hydrophobic core and caused the associated structural disturbance. This structural change potentially influenced the overall protein folding and stability (Figure 4A‐1).

Among the other 11 missense variants, the following 3 variants met the criterion of confidence level >0.6‐*PKD1*: c.4343C > T (p.S1448F), *PKD1*: c.6018G > C (p.W2006C) and *PKD1*: c.6283G > T (p.D2095Y). As depicted in Figure 4A‐2, the side chain of Ser^1448^ formed hydrogen bonds with Arg^1441^, and p.S1448F, which caused the polar serine to be replaced with a large hydrophobic phenylalanine residue, eliminated these hydrogen bonds (Figure 4B‐2). Try^2006^ formed a hydrophobic core with Phe^2008^, Ile^2038^, Val^2040^ and Leu^2053^, and W2006C, by replacing the large benzene ring–containing amino acid with a small and polar cysteine, could destroy this hydrophobic core, thereby causing the associated structural changes and ultimately influencing the overall protein folding and stability (Figure 4A‐3). Asp^2095^ formed hydrogen bonds with Gly^2097^, and the replacement of Asp^2095^ with a tyrosine could eliminate the hydrogen bonds and potentially alter the protein distribution (Figure 4A‐4).

According to the trajectory of RMSD (root mean square deviation) and RMSF (root mean square fluctuation), the p.S1448F, p.W2006C and p.D2095Y variants exhibited higher flexibility compared to the wild‐type (WT) PKD1 (Figure 4B‐1,2; 4C‐1,2; 4D‐1,2). Moreover, the differences among the secondary structures of p.S1448F, p.W2006C, p.D2095Y and WT PKD1 were quite obvious (Figure 4B‐3; 4C‐3; 4D‐3). p.S1448F influenced the formation of the helix and p.D2095Y influenced the sheet structure by altering the hydrogen bonds with Asp^2095^ (Figure 4D‐4).

## 4 DISCUSSION

Over the past few years, different renal polycystic disorders with similar structural alterations have been detected in divergent diseases that include six forms of ADPKD (*PKD1, PKD2, GANAB, DNAJB11*) and ARPKD (*PKHD1*, *DZIP1L*), 15 types of MKS, medullary cystic kidney diseases 1 and 2 (*MUC1* and *MCKD2*), CYSRD, congenital anomalies of the kidney and urinary tract 1‐3 (CAKUT1‐3), polycystic liver disease 1‐4 (PCLD1‐4) and nephronophthisis 1‐17. In these conditions, polycystic deformities may develop at different ages. Autosomal dominant PKDs usually occur in adults, although early development and even in utero*‐*onset have been reported.^11^, ^12^ The variants of co‐factorial genes drastically influence the phenotypes of PKD patients.^35^, ^36^ In the case of no family history of the disease, the decision to straightaway perform target gene analysis becomes difficult.

Ten years ago, Rossetti and colleagues proposed a plausible protocol based on HPLC, LR‐PCR, and direct sequencing to overcome the spurious amplification of the *PKD1* pseudogenes with a detection rate of 63%.^37^ Next‐generation sequencing techniques, either in a targeted sequencing approach^8^, ^11^ or in a more comprehensive manner,^38^ have greatly accelerated variant identification and clinical management.^39^ In order to fulfil the clinical requirements efficiently, a stepwise workflow was established, which included WES and *PKD1*‐targeted LR‐PCR sequencing of exons 1‐34 as the first tier of tests in the screening for possible pathogenic variants for both adult‐ and early‐onset PKDs despite familial history. The main advantage of the proposed experimental detection system was that it combined the advantages of ‘LR‐PCR + Sanger sequencing’ and ‘WES’. Among these two techniques, the former is capable of excluding the pseudogene interference homologous to the *PKD1* gene and ensure the accuracy of the detection of the gene, while the latter considers the comprehensiveness of detecting the variation in the coding region of the whole genome. Using this efficient workflow, the genetic reports of different types of PKDs were completed within 3 weeks and a detection rate of up to 88.5% (23/26) was achieved.

Pathogenic variants in *PKD1* contribute to 85% of the ADPKD cases.^12^ So far, 2072 constitutional pathogenic *PKD1* variants are indexed in the Human Gene Mutation Database (HGMD), spanning the whole gene with no apparent clustering or hot spots. In the present study, 13 novel *PKD1* variants were identified, thereby expanding the variation spectrum of this gene.

The complex of polycystin‐1 (PC1, encoded by *PKD1*) and polycystin‐2 (encoded by *PKD2*) regulates multiple cellular functions, including proliferation, apoptosis, calcium signalling and fluid secretion.^40^ Polycystin‐1 is a transmembrane protein that comprises 4303 amino acids with 11 membrane‐spanning segments.^41^ The overall structural function of PC1 remains elusive to date.^42^ In the present study, the impact of three PKD1 missense variants on the structure of PC1 was analysed, and the results demonstrated that the variants exerted an adverse effect on the structure of the protein and also possibly caused damage to its function that facilitated pathogenicity. Among the three variants, the MD analysis results for *PKD1*: c.9789G > T (p.W3263C) demonstrated that the structural change potentially influenced the overall protein folding and stability.

The cases of early‐onset PKDs have been a concern for decades. The six cases of in utero‐onset PKD included in the present study demonstrated a scenario of clinical similarity and genetic diversity. Five positive families presented various pathogenic traits. Family 21 presented a rare type of biallelic ADPKD of the compound heterozygous *PKD1* gene. The combined effects of the two *PKD1* alleles resulted in a severe foetal‐onset PKD resembling the autosomal recessive ciliopathy syndromes, which was consistent with Al‐Hamed and colleagues'^43^ ‘two‐hit’ theory. Biallelic traits were not detected in Family 15 and Family 19, where both parents were phenotypic PKD patients. These clinical findings indicate that biallelic *PKD1* traits are lethal, as reported by a study conducted with homozygous knock‐out mice.^44^ In the present study, 3 cases (families 4, 10 and 16) with double pathogenic variants in a single allele were detected, although to establish their combined impact on PKD pathogenesis, further molecular and pathological experiments would be required.

Family 22 presented a case of foetal‐onset digenic PKD, in which the foetus carried one monoallelic variant in *PKD1* and two compound heterozygous variants in *PKHD1*. Although the variations of *PKHD1* gene have a high MAF, we concerned about the impact of the variations of *PKHD1* gene in combination with family and clinical data. Two previous mouse studies have reported homozygous *PKHD1* variants in addition to either a monoallelic *PKD1* or *PKD2* variant resulting in severe phenotypes and embryonic lethality.^45^, ^46^ Therefore, we hypothesized that the combinational effects of the ADPKD and ARPKD traits having been proposed to produce further severe and early‐onset phenotypes. The genetic experiment results in the present study provided probable evidence in favour of phenotypic amplification in the digenic patient. Follow‐up functional experiments are needed to elucidate the pathogenic mechanism of this case.

Family 24 and Family 25 were screened out using prenatal imaging diagnosis for foetal PKD, although additional syndromic manifestations appeared in Family 25. The genetic diagnosis for both these families was MKS consistent with autosomal recessive (AR) patterns. Biallelic pathogenic variants in the *TMEM67* gene may lead to MKS3, with overlapping symptoms to ADPKD, including renal cysts and central nervous dysplasia, among which the latter was not evidently manifested in Family 24. One possible explanation for this could be that c.637C > T (p.R213C) was possibly a hypomorphic variant.^47^ The *TMEM107* gene is expressed in a subset of embryonic tissues^48^ and is involved in cilia formation. This gene was designated as the causative gene for MKS13.^49^ The proband foetus with typical MKS13 phenotypes in Family 25 carried two novel *TMEM107* variants: c.129delC (p.E44Rfs*98) and c.64_66delGTC (p.V22del), both of which were predicted with large fragment truncation and were expected to cause a functional defect.

In the final report, two cases of adult‐onset (families 19 and 20) and one case of foetal‐onset (Family 26) presented negative results. The causes might include mosaicism, variants outside the exons, inversion and epigenetic change, among others. Recent studies have indicated that *PKD1* mosaicism occurring in the early‐stage embryos might respond to a few of the unsolved cases^50^ and could explain the intra‐familial phenotype variability.^51^, ^52^ Nonetheless, Family 26 demonstrated clinical foetal‐onset and other syndromic indications (Figure S1). Further detailed CNV analysis and whole‐genome sequencing may facilitate the identification of diverse noncoding variants and tissue mosaicism.

Since the sample size of the present study was small, the statistical genotype‐phenotype correlations in PKD could not be established. With the development and progress of genetic databases and bioinformatics tools and techniques, it is expected that there would be a large number of phenotypic prediction methods available for guiding the clinical genetic management based on the overall cystogene background of individuals.^1^

## 5 CONCLUSION

In the present study, a comprehensive genetic strategy was developed to identify genomic variants in 26 PKD families. A total of 32 variants were identified in 23 families, among which 16 were the novel ones. The present study expanded the variation spectrum of cystogenes, and in particular, it revealed further solid evidence for foetal‐onset PKDs usually occurring with biallelic and digenic variants. The biophysical analysis of the missense variants in the *PKD1* gene illuminated a potential tool to assist in the determination of pathogenicity.

## CONFLICT OF INTEREST

The authors declare no conflict of interest.

## AUTHOR CONTRIBUTION

**Hua‐Ying Hu:** Conceptualization (equal); Data curation (equal); Formal analysis (equal); Investigation (equal); Methodology (equal); Software (equal); Writing‐original draft (equal). **Jing Zhang:** Conceptualization (equal); Resources (equal). **Wei Qiu:** Resources (equal); Validation (equal). **Chao Liang:** Resources (equal). **Cun‐Xi Li:** Writing‐review & editing (equal). **Tian‐Ying Wei:** Data curation (equal); Validation (equal). **Zhan‐Ke Feng:** Data curation (equal); Validation (equal). **Qing Guo:** Conceptualization (equal); Project administration (equal); Resources (equal). **Kai Yang:** Software (equal); Writing‐review & editing (equal). **Zu‐Guo Liu:** Conceptualization (equal); Project administration (equal); Writing‐review & editing (equal).

## Supporting information

Figure S1‐1

Figure S1‐2

Figure S1‐3

Figure S1‐4

Figure S2

Figure S3

Table S1

Table S2

Table S3

## Data Availability

The data that support the findings of this study are available from the corresponding author upon reasonable request.
