# Supplementary figures and images for: Comprehensive strategy improves the genetic diagnosis of different polycystic kidney diseases
Source: J Cell Mol Med. 2021 May 25;25(13):6318–32. doi: 10.1111/jcmm.16608 (PMC8256360; doi:10.1111/jcmm.16608)

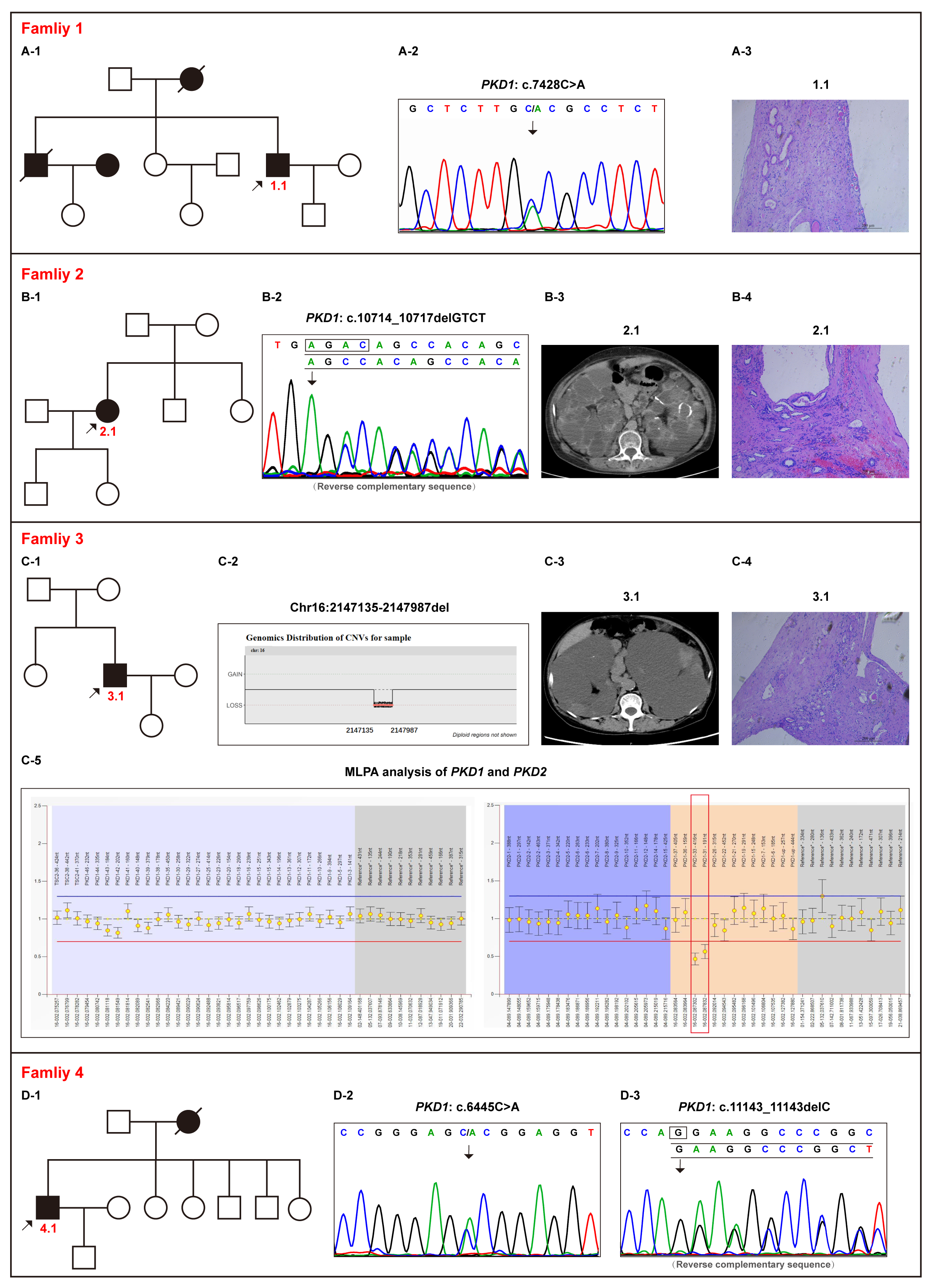

Supplement: Supplementary file 1 — Figure S1‐1 [file JCMM-25-6318-s006.tif]

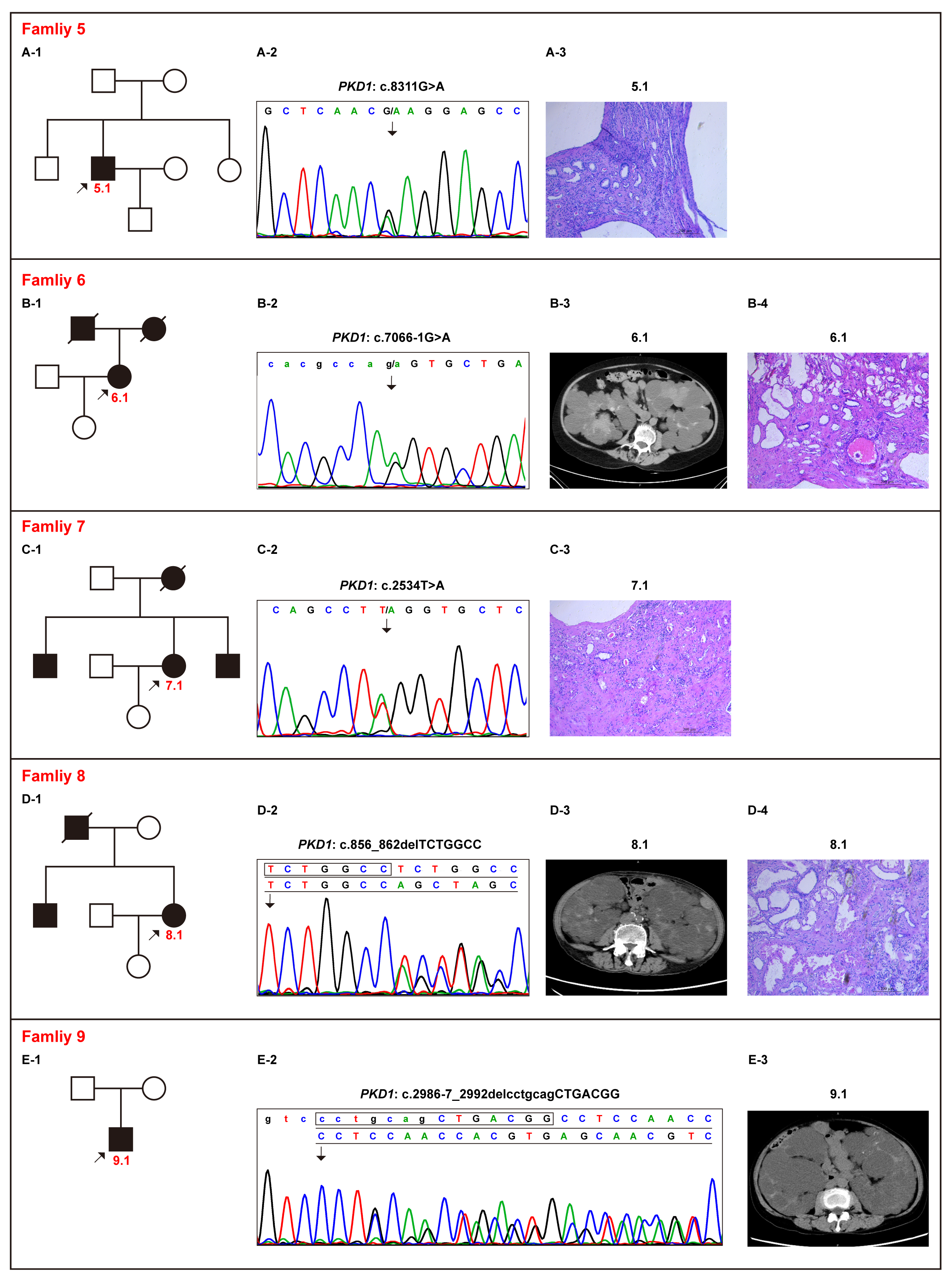

Supplement: Supplementary file 2 — Figure S1‐2 [file JCMM-25-6318-s004.tif]

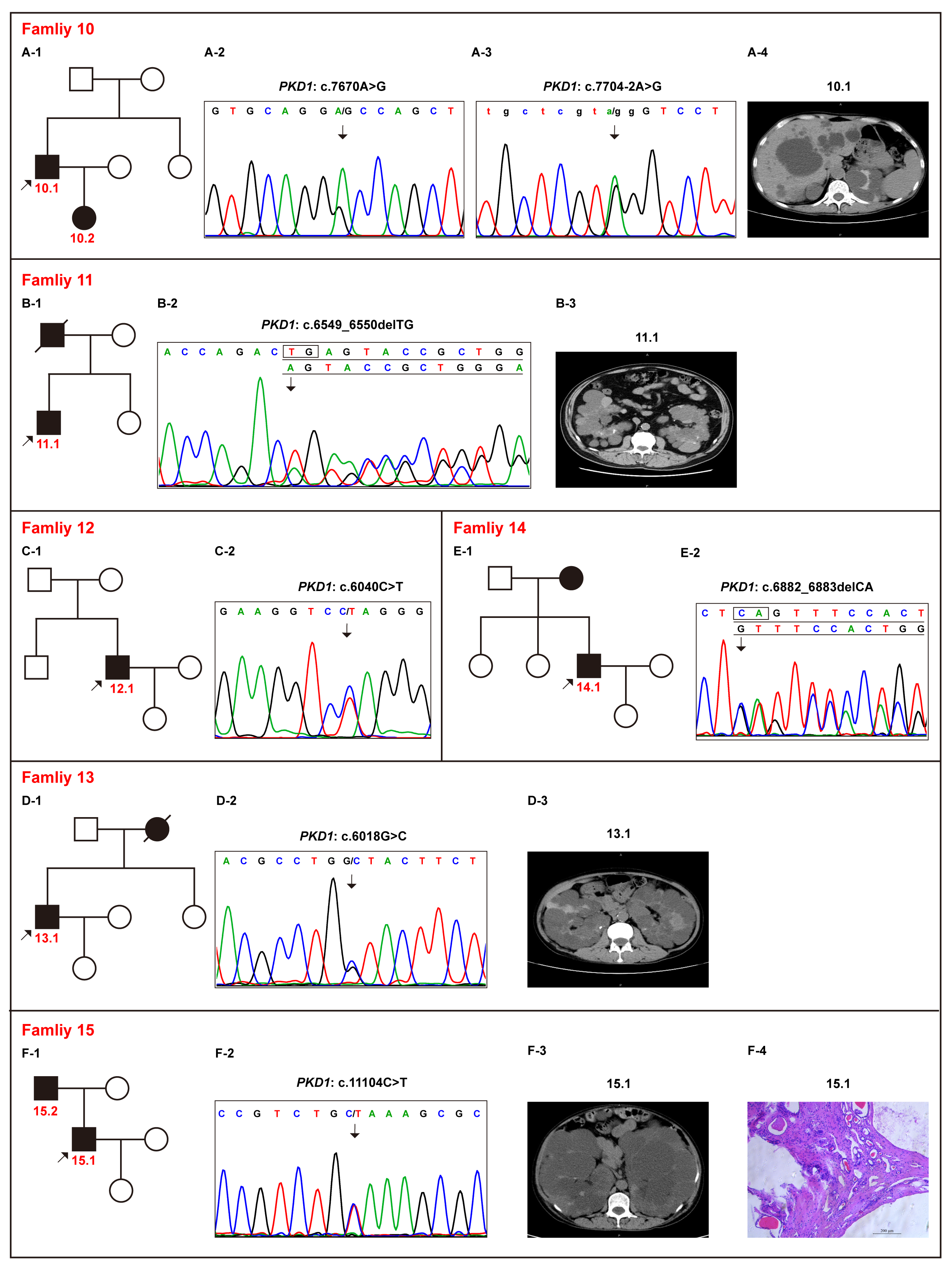

Supplement: Supplementary file 3 — Figure S1‐3 [file JCMM-25-6318-s007.tif]

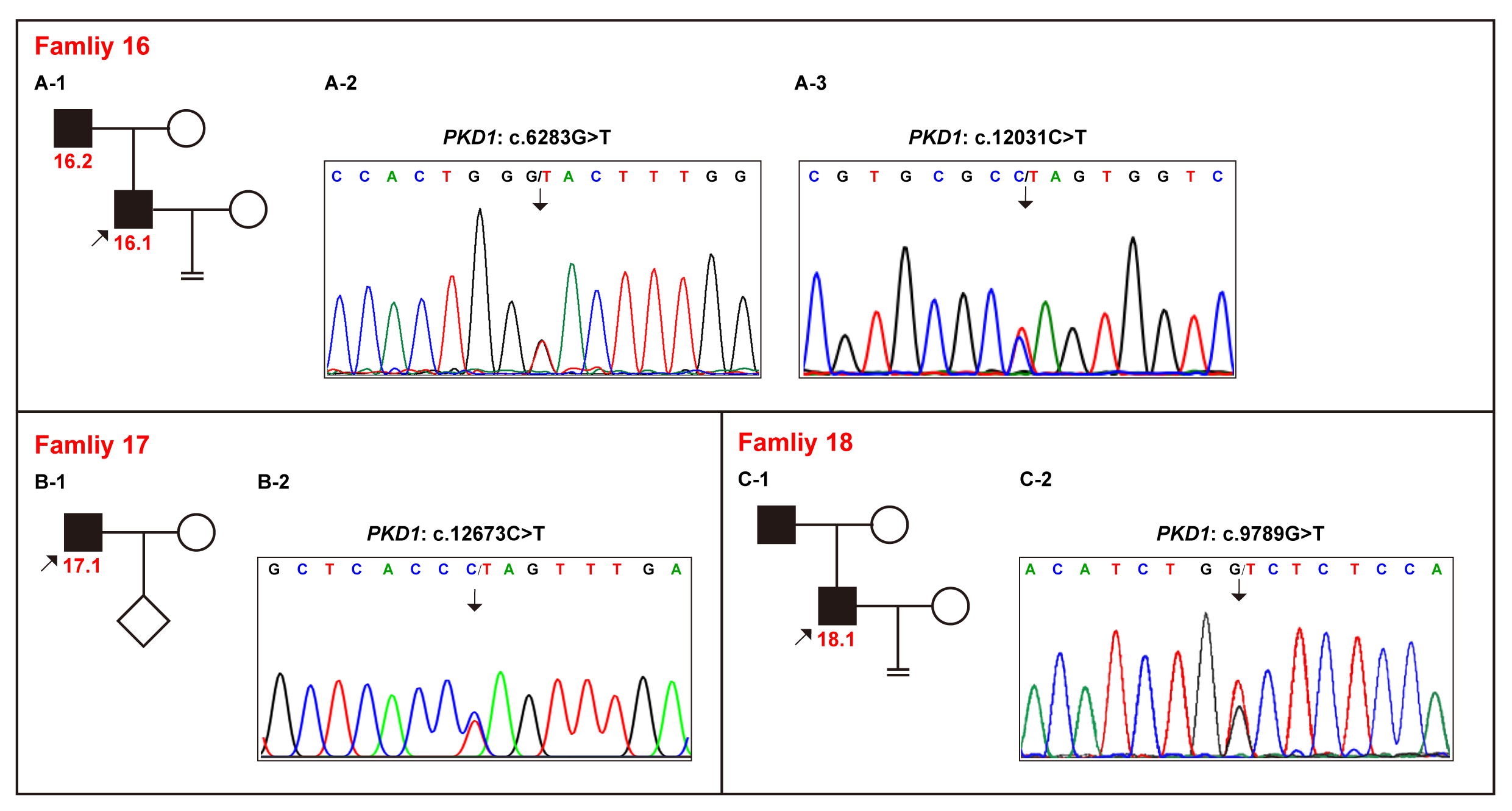

Supplement: Supplementary file 4 — Figure S1‐4 [file JCMM-25-6318-s009.tif]

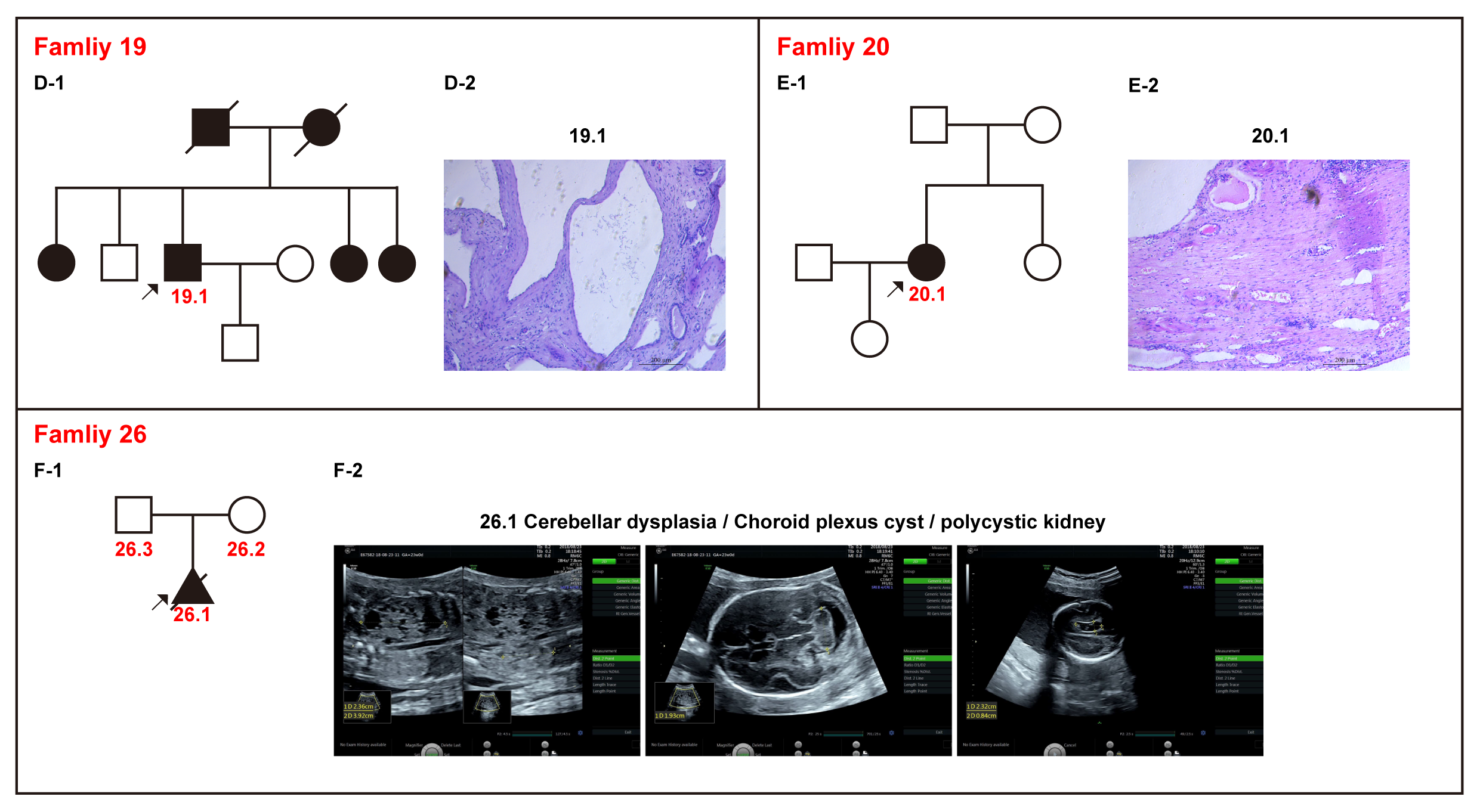

Supplement: Supplementary file 5 — Figure S2 [file JCMM-25-6318-s008.tif]

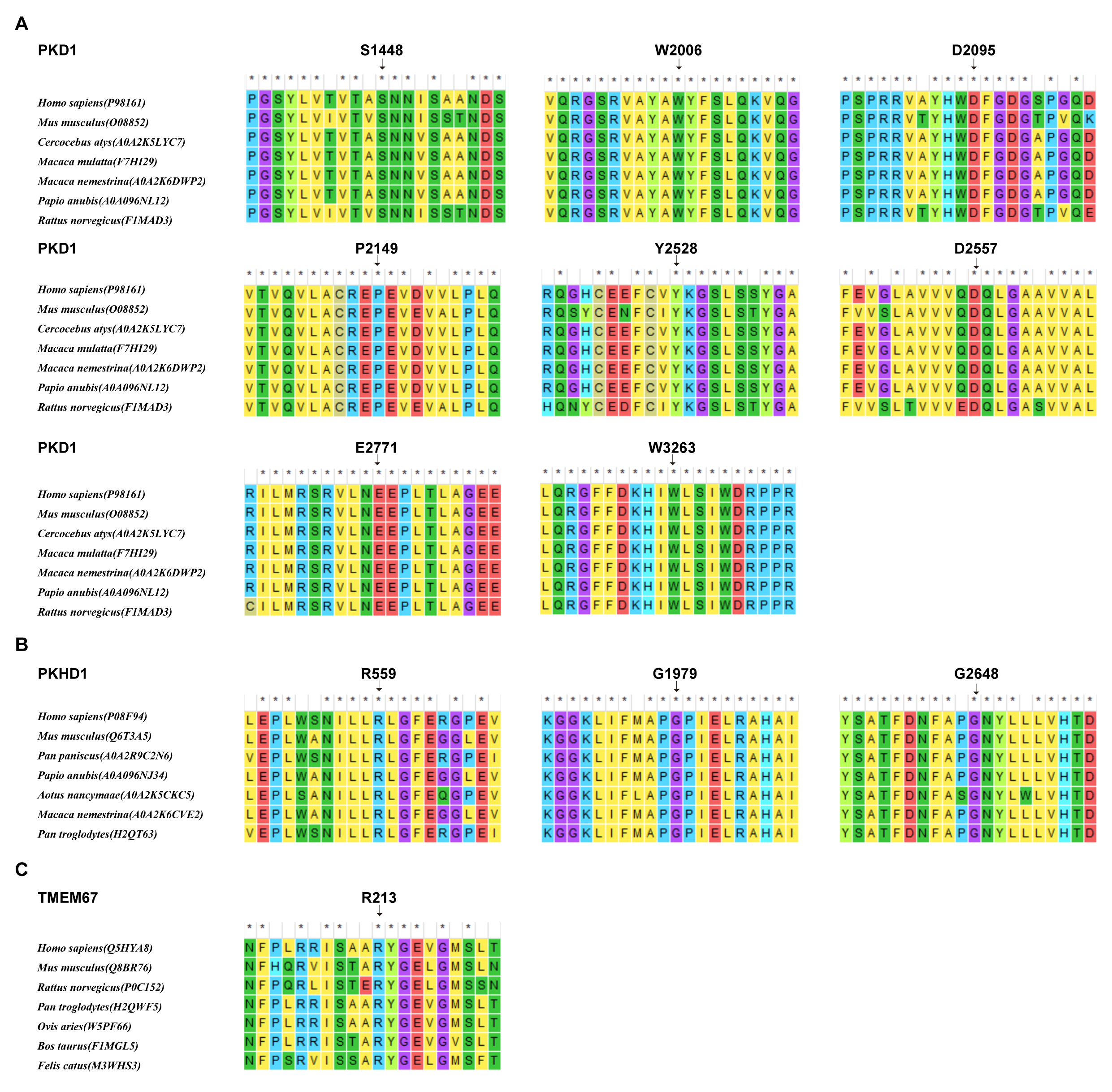

Supplement: Supplementary file 6 — Figure S3 [file JCMM-25-6318-s001.tif]
