# Supplementary material for: Comprehensive strategy improves the genetic diagnosis of different polycystic kidney diseases
Source: J Cell Mol Med. 2021 May 25;25(13):6318–32. doi: 10.1111/jcmm.16608 (PMC8256360; doi:10.1111/jcmm.16608)
Supplement: Supplementary file 7 — Table S1 [file JCMM-25-6318-s002.docx]

**Table S1. The panel of 313 genes associated with different polycystic kidney diseases**

| *ACTG2* | *BUB1B* | *DNAJC10* | *HNF1B* | *MKKS* | *PEX11B* | *SEC31A* | *TMEM237* |
| --- | --- | --- | --- | --- | --- | --- | --- |
| *ACE* | *C5orf42* | *DNAJC3* | *HNF4A* | *MKS1* | *PEX12* | *SEC61A1* | *TMEM260* |
| *ACTN4* | *C8orf37* | *DYNC2H1* | *HOXD13* | *MKS3* | *PEX13* | *SEC61A2* | *TMEM67* |
| *AGXT* | *CA2* | *DZIP1L* | *HPSE2* | *MLEC* | *PEX14* | *SEC61B* | *TRAF3IP1* |
| *AHI1* | *CALR* | *EDEM1* | *HSP90B1* | *MOGS* | *PEX16* | *SEC61G* | *TRIM21* |
| *ALG8* | *CANX* | *EDEM2* | *HSPA5* | *MSTN* | *PEX19* | *SEC62* | *TRIM32* |
| *ALG9* | *CC2D2A* | *EDEM3* | *HYOU1* | *MT-TL1* | *PEX2* | *SEC63* | *TRIP11* |
| *AMER1* | *CCDC28B* | *EFCAB7* | *IFT122* | *MUC1* | *PEX26* | *SETD5* | *TRIP13* |
| *ANKS6* | *CD2AP* | *EIF2AK3* | *IFT140* | *MYH11* | *PEX3* | *SHANK3* | *TRPC6* |
| *APRT* | *CD96* | *ERLEC1* | *IFT144* | *MYLK* | *PEX5* | *SIL1* | *TSC1* |
| *ARL13B* | *CDC5L* | *ERO1A* | *IFT172* | *NEK1* | *PEX6* | *SIX1* | *TSC2* |
| *ARL3* | *CDC73* | *ERO1LB* | *IFT27* | *NEK8* | *PIEZO2* | *SIX5* | *TTBK2* |
| *ARL6* | *CDKN1C* | *ESCO2* | *IFT43* | *NFIA* | *PIGN* | *SKIV2L* | *TTC21B* |
| *ATF6B* | *CEP164* | *ETFA* | *IFT74* | *NIPBL* | *PIGQ* | *SLC12A1* | *TTC37* |
| *ATP2A2* | *CEP290* | *ETFB* | *IFT80* | *NOTCH2* | *PKD1* | *SLC12A3* | *TTC8* |
| *ATP6V0A4* | *CEP41* | *ETFDH* | *IGF2* | *NPHP1* | *PKD2* | *SLC3A1* | *UGGT1* |
| *ATP6V1B1* | *CEP55* | *EYA1* | *INF2* | *NPHP10* | *PKHD1* | *SLC4A1* | *UGGT2* |
| *ATXN10* | *CEP57* | *FAM58A* | *INPP5E* | *NPHP2* | *PLCE1* | *SLC5A1* | *UMOD* |
| *AVPR2* | *CEP83* | *FCYT* | *INVS* | *NPHP3* | *PMM2* | *SLC5A2* | *UPK3A* |
| *B3GLCT* | *CHRM3* | *FIBP* | *IQCB1* | *NPHP4* | *PORCN* | *SLC7A9* | *USF2* |
| *B9D1* | *CKAP4* | *FLCN* | *JBTS11* | *NPHP5* | *PRKAR1A* | *SLC9A3R1* | *VHL* |
| *B9D2* | *CLCN5* | *FLI1* | *KAT6B* | *NPHP6* | *PRKCSH* | *SMARCAL1* | *WDPCP* |
| *BB16* | *CLDN16* | *FLNC* | *KCNJ1* | *NPHP7* | *PTHB1* | *SMC1A* | *WDR19* |
| *BBIP1* | *COL4A1* | *FRAS1* | *KCNQ1* | *NPHP8* | *RAD21* | *SMC3* | *WDR34* |
| *BBS1* | *COL4A2* | *FREM2* | *KCNQ1OT1* | *NPHP9* | *REN* | *SNRPB* | *WDR35* |
| *BBS10* | *COL4A3* | *FXYD2* | *KIAA0586* | *NPHS1* | *RET* | *SOX17* | *WNK1* |
| *BBS12* | *COL4A4* | *GANAB* | *KIF7* | *NPHS2* | *ROR2* | *SPIN4* | *WNK4* |
| *BBS18* | *COL4A5* | *GATA3* | *KMT2A* | *NR3C2* | *RPGRIP1* | *STT3A* | *WNT3* |
| *BBS19* | *COL4A6* | *GLA* | *LAMA3* | *NRIP1* | *RPGRIP1L* | *STT3B* | *WT1* |
| *BBS2* | *COMT* | *GLIS2* | *LAMB2* | *NXF5* | *RPN1* | *TBCK* | *XDH* |
| *BBS4* | *COQ2* | *GLIS3* | *LAMB3* | *NXN* | *RPN2* | *TBX1* | *XPNPEP3* |
| *BBS5* | *CPT2* | *GP1BB* | *LAMC2* | *OCRL* | *RSPO2* | *TBX18* | *ZEB2* |
| *BBS6* | *CSPP1* | *GPC3* | *LIPT2* | *OFD1* | *SALL1* | *TCTN1* | *ZNF423* |
| *BBS7* | *CTNS* | *GPC4* | *LMX1B* | *OS9* | *SCARB2* | *TCTN2* |  |
| *BBS8* | *DCDC2* | *GREB1L* | *LPIN1* | *PAX2* | *SCNN1A* | *TCTN3* |  |
| *BBS9* | *DDOST* | *GRHPR* | *LRP2* | *PDE6D* | *SCNN1B* | *TFAP2A* |  |
| *BICC1* | *DHCR7* | *GRIP1* | *LRP5* | *PDIA3* | *SDCCAG8* | *TMEM107* |  |
| *BNC2* | *DNAJB11* | *H19-ICR* | *LZTFL1* | *PDSS2* | *SEC13* | *TMEM138* |  |
| *BSND* | *DNAJB9* | *HDAC8* | *MAPKBP1* | *PEX1* | *SEC24B* | *TMEM216* |  |
