# Supplementary material for: Comprehensive strategy improves the genetic diagnosis of different polycystic kidney diseases
Source: J Cell Mol Med. 2021 May 25;25(13):6318–32. doi: 10.1111/jcmm.16608 (PMC8256360; doi:10.1111/jcmm.16608)
Supplement: Supplementary file 8 — Table S2 [file JCMM-25-6318-s003.docx]

**Table S2. PCR Primers used in LR-PCR and Sanger sequencing**

| **Fragment** | **Primers** | **Sequence** | **Size** | **Tm** | **Experiment system^#^** |
| --- | --- | --- | --- | --- | --- |
| PKD1_Ex1 | PKD1-LR-Ex1-F | 5'-CGCAGCCTTACCATCCACCT-3' | 2.3kb | 68℃ | GXL |
|  | PKD1-LR-Ex1-R | 5'-TCATCGCCCCTTCCTAAGCA-3' |  |  |  |
| PKD1_Ex2-12 | PKD1-LR-Ex2-12-F | 5'-CCAGCTCTCTGTCTACTCACCTCCGCATC-3' | 8.7kb | 68℃ | GXL |
|  | PKD1-LR-Ex2-12-R | 5'-CCACGGTTACGTTGTAGTTCACGGTGACG-3' |  |  |  |
| PKD1_Ex13-21 | PKD1-LR-Ex13-21-F | 5'-TGGAGGGAGGGACGCCAATC-3' | 7.9kb | 68℃ | GXL |
|  | PKD1-LR-Ex13-21-R | 5'-ACACAGGACAGAACGGCTGAGGCTA-3' |  |  |  |
| PKD1_Ex22-34 | PKD1-LR-Ex22-34-F | 5'-ATGCTTAGTGAGGAGGCTGTGGGGGTC-3' | 7.8kb | 68℃ | GXL |
|  | PKD1-LR-Ex22-34-R | 5'-ATGAGGCTCTTTCCACAGACAACAGAGGTT-3' |  |  |  |
| PKD1-exon1 | PKD1-exon1-F2 | 5'-CAGTCCCTCATCGCTGGC-3' | 699bp | 68℃ | HS |
|  | PKD1-exon1-R2 | 5'-TCCTTATTTAGCAGGGCCGC-3' |  |  |  |
| PKD1-exon2-3 | PKD1-exon2-3-F | 5'-CCTCTCCTGGCTTGGTTTCC-3' | 536bp | 62℃ | TAQ |
|  | PKD1-exon2-3-R | 5'-CAACTGGGAGGGCAGAAGG-3' |  |  |  |
| PKD1-exon4 | PKD1-exon4-F2 | 5'-CCAGGCTTGAGACCAGATCC-3' | 496bp | TD | TAQ |
|  | PKD1-exon4-R2 | 5'-CCGCCGCACTCACAGG-3' |  |  |  |
| PKD1-exon5 | PKD1-exon5-F4 | 5'-CGGATGTGGGGGCGTG-3' | 839bp | 70℃ | HS |
|  | PKD1-exon5-R4 | 5'-GAGGGTGTCAACGGTCAGTG-3' |  |  |  |
| PKD1-exon6 | PKD1-exon6-F2 | 5'-CGACCTCAGCATCCAGAACC-3' | 570bp | TD | HS |
|  | PKD1-exon6-R2 | 5'-CCAGCTCATGTCCACCTCTG-3' |  |  |  |
| PKD1-exon7-8 | PKD1-exon7-8-F | 5'-GCCCTGGTCTCCGGG-3' | 749bp | 62℃ | TAQ |
|  | PKD1-exon7-8-R | 5'-TTTTGGCGAGACCCACAGT-3' |  |  |  |
| PKD1-exon9 | PKD1-exon9-F | 5'-TCTCCTTCCCTCCCCTCTTC-3' | 549bp | 62℃ | TAQ |
|  | PKD1-exon9-R | 5'-CACCGTCAGAGATGCCCAAC-3' |  |  |  |
| PKD1-exon10 | PKD1-exon10-F | 5'-CTCACAGCAAGGCCAGGATT-3' | 486bp | 62℃ | TAQ |
|  | PKD1-exon10-R | 5'-AGGTCGGAGGTCAGAGGTG-3' |  |  |  |
| PKD1-exon11 | PKD1-exon11-1F | 5'-TTGAGGTGTGGCTGACGAAG-3' | 589bp | 62℃ | TAQ |
|  | PKD1-exon11-1R | 5'-TGGCCGTGGCGTTGG-3' |  |  |  |
| PKD1-exon11 | PKD1-exon11-2F | 5'-CCCAACCCTGGACTGCG-3' | 597bp | 62℃ | TAQ |
|  | PKD1-exon11-2R | 5'-TGCTGCCCTCACTGGGAA-3' |  |  |  |
| PKD1-exon12 | PKD1-exon12-F2 | 5'-CTACATGGCTGGCTGGAGAG-3' | 417bp | TD | HS |
|  | PKD1-exon12-R2 | 5'-AAGCAGAGCAGAAGGCAGAG-3' |  |  |  |
| PKD1-exon13 | PKD1-exon13-F4 | 5'-TTCACCTCTGCCTTCTGCTC-3' | 453bp | TD | TAQ |
|  | PKD1-exon13-R4 | 5'-CTCCTGTGCACCCAGTTACC-3' |  |  |  |
| PKD1-exon14 | PKD1-exon14-F4 | 5'-CATCACGGGGTTGCTTTTCT-3' | 561bp | TD | TAQ |
|  | PKD1-exon14-R4 | 5'-CCAGCTTCCCTGTCCACTC-3' |  |  |  |
| PKD1-exon15 | PKD1-exon15-1F | 5'-GCTTCTGCCGAGCGGGTG-3' | 639bp | 60℃ | TAQ |
|  | PKD1-exon15-1R | 5'-ACGTGCAGGCTCCGGG-3' |  |  |  |
|  | PKD1-exon15-2F | 5'-GCAACAGTGGAGCATGTGTA-3' | 650bp | 60℃ | TAQ |
|  | PKD1-exon15-2R | 5'-TTGACCTTGATGCTGGTGAC-3' |  |  |  |
|  | PKD1-exon15-3F | 5'-ACAGTCACCGCGTCCA-3' | 646bp | 60℃ | TAQ |
|  | PKD1-exon15-3R | 5'-CATCCCTAACCACGGCCT-3' |  |  |  |
|  | PKD1-exon15-4F | 5'-AGCATCTTCGTCTATGTCCTGC-3' | 650bp | 60℃ | TAQ |
|  | PKD1-exon15-4R | 5'-CAGCACCAGCTCACATTGGT-3' |  |  |  |
|  | PKD1-exon15-5F | 5'-CATCAGGGCCAGCGAGC-3' | 649bp | TD | TAQ |
|  | PKD1-exon15-5R | 5'-CGACAGGATGACCAGCGAG-3' |  |  |  |
|  | PKD1-exon15-6F | 5'-ACTTCACAGCCCGCGT-3' | 650bp | TD | TAQ |
|  | PKD1-exon15-6R | 5'-GGGCAGGGCCACACG-3' |  |  |  |
|  | PKD1-exon15-7F | 5'-CAACTACTTGGAGGCCCACG-3' | 594bp | TD | TAQ |
|  | PKD1-exon15-7R | 5'-CCAGTTTTAAAGCAGAGCCCG-3' |  |  |  |
| PKD1-exon16 | PKD1-exon16-F | 5'-CCATGAGCCCAGAGAACACC-3' | 436bp | 62℃ | TAQ |
|  | PKD1-exon16-R | 5'-AAACAGAGAGGGGAGAGCGT-3' |  |  |  |
| PKD1-exon17 | PKD1-exon17-18-F | 5'-CATGGGTCCCCCAGTCCT-3' | 700bp | TD | TAQ |
|  | PKD1-exon17-18-R | 5'-CCCACGGCATCACGGG-3' |  |  |  |
| PKD1-exon19-20 | PKD1-exon19-20-F | 5'-GGGGGAGCAGCGGGAT-3' | 586bp | 62℃ | TAQ |
|  | PKD1-exon19-20-R | 5'-AGGCAGGGGTACAGGTCTT-3' |  |  |  |
| PKD1-exon21 | PKD1-exon21-F | 5'-GCACCCCGACCTTCCGCTC-3' | 590bp | 62℃ | TAQ |
|  | PKD1-exon21-R | 5'-ACACAGGACAGAACGGCTGAGGCTA-3' |  |  |  |
| PKD1-exon22 | PKD1-exon22-F | 5'-GTGAGGACCCGTGTAGAGAG-3' | 326bp | 66℃ | HS |
|  | PKD1-exon22-R | 5'-GAAGATGGGATGGGGCAAAG-3' |  |  |  |
| PKD1-exon23 | PKD1-exon23-F | 5'-CACTCTCTCTCCAGCTTCCC-3' | 871bp | 66℃ | HS |
|  | PKD1-exon23-R | 5'-CCATGGAAGCCCTACGAGAA-3' |  |  |  |
| PKD1-exon24-25 | PKD1-exon24-25-F | 5'-GAGGCTCCTATGGCCTTTCAG-3' | 787bp | 62℃ | TAQ |
|  | PKD1-exon24-25-R | 5'-GTGCCTTCTCAGGATAGAGCC-3' |  |  |  |
| PKD1-exon26 | PKD1-exon26-F | 5'-GAGTGACCCTGTGCTCCTG-3' | 434bp | 62℃ | TAQ |
|  | PKD1-exon26-R | 5'-CAGCACTGCAAAAACTGCCT-3' |  |  |  |
| PKD1-exon27-28 | PKD1-exon27-28-F | 5'-AGTGCGCACAGGCCAAA-3' | 569bp | 62℃ | TAQ |
|  | PKD1-exon27-28-R | 5'-CACTGCAGGAGGCCACG-3' |  |  |  |
| PKD1-exon29-30 | PKD1-exon29-30-F | 5'-TCGTTCCATGTTCCCACTCC-3' | 598bp | 62℃ | TAQ |
|  | PKD1-exon29-30-R | 5'-AGCACTGGAAAGTGGCGG-3' |  |  |  |
| PKD1-exon31-32 | PKD1-exon31-32-F | 5'-GAGGTGGCTTCTCATCCCTG-3' | 469bp | TD | TAQ |
|  | PKD1-exon31-32-R | 5'-GGGCTTCCGAGCAAACCTG-3' |  |  |  |
| PKD1-exon33-34 | PKD1-exon33-34-F2 | 5'-GGGTGTCCGTGCGTGACTGGA-3' | 614bp | TD | TAQ |
|  | PKD1-exon33-34-R2 | 5'-GGATCCCATGAGGCTCTTTCCACAG-3' |  |  |  |
| PKD1-exon35-36 | PKD1-exon35-36F | 5'-AGGTTAACATGGGCTTGGCT-3' | 542bp | TD | TAQ |
|  | PKD1-exon35-36R | 5'-GGGGAGGGGGTGGCTTC-3' |  |  |  |
| PKD1-exon37 | PKD1-exon37-F | 5'-GGGGCTGCCAGGGGTA-3' | 379bp | TD | TAQ |
|  | PKD1-exon37-R | 5'-AACCCCTAAGGGCCTTCTGA-3' |  |  |  |
| PKD1-exon38 | PKD1-exon38-F | 5'-GTGTGTGCTGCCATTACCCT-3' | 422bp | TD | TAQ |
|  | PKD1-exon38-R | 5'-CCTCCAGTTCTAGCAGCCAC-3' |  |  |  |
| PKD1-exon39-40 | PKD1-exon39-40-F | 5'-CTGGCATCAGTAGGCAGAGG-3' | 460bp | TD | TAQ |
|  | PKD1-exon39-40-R | 5'-AGAGGGCAAAGGTCACACAG-3' |  |  |  |
| PKD1-exon41-42 | PKD1-exon41-42-F2 | 5'-GCCAAGGACAAGGGAGTAGT-3' | 686bp | TD | HS |
|  | PKD1-exon41-42-R2 | 5'-TGCGAGGGGTGAGACG-3' |  |  |  |
| PKD1-exon43-44 | PKD1-exon43-44-F4 | 5'-GCAGCGTCTCACCCCT-3' | 777bp | 68℃ | HS |
|  | PKD1-exon43-44-R4 | 5'-ACACAGGAAGACACGAGCTG-3' |  |  |  |
| PKD1-exon45 | PKD1-exon45-F | 5'-TCTTTGGCAAGACATTATGCCG-3' | 590bp | 60℃ | TAQ |
|  | PKD1-exon45-R | 5'-CTTTGTGGCGGAACTGGGG-3' |  |  |  |
| PKD1-exon46 | PKD1-exon46-F | 5'-GCCTCAGCAAGGTCAAGGAG-3' | 686bp | 62℃ | TAQ |
|  | PKD1-exon46-R | 5'-AACCTACGTGCAGCCATTCT-3' |  |  |  |
| TMEM67-exon7 | TMEM67-exon7-F | 5'-TGAACAGGTGCGTCCGATG-3' | 353bp | 60℃ | TAQ |
|  | TMEM67-exon7-R | 5'-AATGATTTACACAAAAGGGTTTCTC-3' |  |  |  |
| TMEM107-exon1 | TMEM107-exon1-F | 5'-TTTTCTTGTTTCCCGCCG | 203bp | 60℃ | TAQ |
|  | TMEM107-exon1-R | 5'-ACCCCTCCACTCCCACCAC-3' |  |  |  |
| TMEM107-exon2 | TMEM107-exon2-F | 5'-AGATTTGTCGGCTTGCGG-3' | 413bp | 60℃ | TAQ |
|  | TMEM107-exon2-R | 5'-ATGTCCTGCTTGTCATACTCCTC-3' |  |  |  |
| PKHD1-exon17-18 | PKHD1- exon17-18-F | 5'-AGGAAATAGCAAGTTGAGGAGGA-3' | 579bp | 60℃ | TAQ |
|  | PKHD1- exon17-18-R | 5'-TATTGACACCCACCGAGCATC-3' |  |  |  |
| PKHD1- exon32 | PKHD1- exon32-F | 5'-TTGCCTGGAGCTTCCTTCTC-3' | 280bp | TD | TAQ |
|  | PKHD1- exon32-R | 5'-GGGCCAAGTCTCTTGTCTGG-3' |  |  |  |
| PKHD1- exon37 | PKHD1- exon37-F | 5'-ACGGTAAGCCTTATCCTCCCA-3' | 391bp | TD | TAQ |
|  | PKHD1- exon37-R | 5'-GGAAGGGCTTGTCTTCGGAT-3' |  |  |  |
| PKHD1- exon50 | PKHD1- exon50-F | 5'-GTGGTGTGGTGGAGTAGGTG-3' | 463bp | 60℃ | TAQ |
|  | PKHD1- exon50-R | 5'-TGCTAGCTGGTGAGTGTTGG-3' |  |  |  |

TD, Touch-down protocol; ^#^: Detail in Table S3
