# Supplementary material for: Comprehensive strategy improves the genetic diagnosis of different polycystic kidney diseases
Source: J Cell Mol Med. 2021 May 25;25(13):6318–32. doi: 10.1111/jcmm.16608 (PMC8256360; doi:10.1111/jcmm.16608)
Supplement: Supplementary file 9 — Table S3 [file JCMM-25-6318-s005.docx]

**Table S3. Protocols for PCR amplifications**

| **Experiment system** | **PCR conditions** | **PCR enzyme and buffer** |
| --- | --- | --- |
| GXL | 98°C, 3 min; followed by 35 cycles: 98°C, 30 sec→60°C, 15 sec→68°C, 1.5 min; final extension at 72°C, 7 min. | TAKARA PrimeSTAR GXL (PrimeSTAR GXL buffer with 0.5M Betaine and 5% DMSO |
| TAQ / HS | **Touch-down** protocol:  The initial step at 95°C, 5 min; followed by 21 cycles: 95°C, 30 sec→70°C, 30 sec, with decreasing 1°C per cycle→72°C, 30 sec; followed by 25 cycles: 95°C, 30 sec→50°C, 30 sec→72°C, 30 sec; final extension at 72°C, 10 min. | TAKARA Ex Taq® /  TAKARA PrimeSTAR® HS DNA Polymerase (with 0.5M Betaine) |
|  | Regular Protocol:  The initial step at 95°C, 5 min; followed by 30 cycles: 95°C, 30 sec→ annealed for 30sec based on their Tm values→72°C, 30 sec; final extension at 72°C, 10 min. |  |
